# Bioinformatic tools for microRNA dissection

**DOI:** 10.1093/nar/gkv1221

**Published:** 2015-11-17

**Authors:** Most Mauluda Akhtar, Luigina Micolucci, Md Soriful Islam, Fabiola Olivieri, Antonio Domenico Procopio

**Affiliations:** 1 Laboratory of Experimental Pathology, Department of Clinical and Molecular Sciences, Università Politecnica delle Marche, Ancona 60100, Italy; 2 Computational Pathology Unit, Department of Clinical and Molecular Sciences, Università Politecnica delle Marche, Ancona 60100, Italy; 3 Department of Experimental and Clinical Medicine, Faculty of Medicine, Università Politecnica delle Marche, Ancona 60100, Italy; 4 Center of Clinical Pathology and Innovative Therapies, Italian National Research Center on Aging (INRCA-IRCCS), Ancona 60121, Italy

## Abstract

Recently, microRNAs (miRNAs) have emerged as important elements of gene regulatory networks. MiRNAs are endogenous single-stranded non-coding RNAs (∼22-nt long) that regulate gene expression at the post-transcriptional level. Through pairing with mRNA, miRNAs can down-regulate gene expression by inhibiting translation or stimulating mRNA degradation. In some cases they can also up-regulate the expression of a target gene. MiRNAs influence a variety of cellular pathways that range from development to carcinogenesis. The involvement of miRNAs in several human diseases, particularly cancer, makes them potential diagnostic and prognostic biomarkers. Recent technological advances, especially high-throughput sequencing, have led to an exponential growth in the generation of miRNA-related data. A number of bioinformatic tools and databases have been devised to manage this growing body of data. We analyze 129 miRNA tools that are being used in diverse areas of miRNA research, to assist investigators in choosing the most appropriate tools for their needs.

## INTRODUCTION

One of the most exciting biological discoveries in the past decade is non-coding RNAs. MicroRNAs (miRNAs) are very small (∼22-nt long), non-protein-coding, single-stranded RNAs that regulate the expression of protein-coding genes (1,2). They comprise a subset of non-coding RNAs that play a key role in gene regulation as part of large and complex gene regulatory networks (3). Most mammalian miRNAs are encoded by RNA Polymerase II (4). MiRNAs are found in different genomic regions: introns of protein-coding genes; exons and introns of non-coding genes (5), and even the 3′ untranslated region (3′ UTR) of protein-coding genes (6). About one-third of mammalian miRNAs are embedded in introns of protein-coding genes and have the same transcription pattern as the protein-coding genes where they reside (5). Over the past few years, their biogenesis has extensively been explored (3,7–9). Based on computational predictions ∼60% of human protein-coding genes are targeted by miRNAs through conserved base-pairing between the 3′ UTR of mRNA and the 5′ region of miRNA, called the seed region (10). Pairing causes inhibition of translation and/or degradation of target mRNAs (3,8).

**Figure 1.**
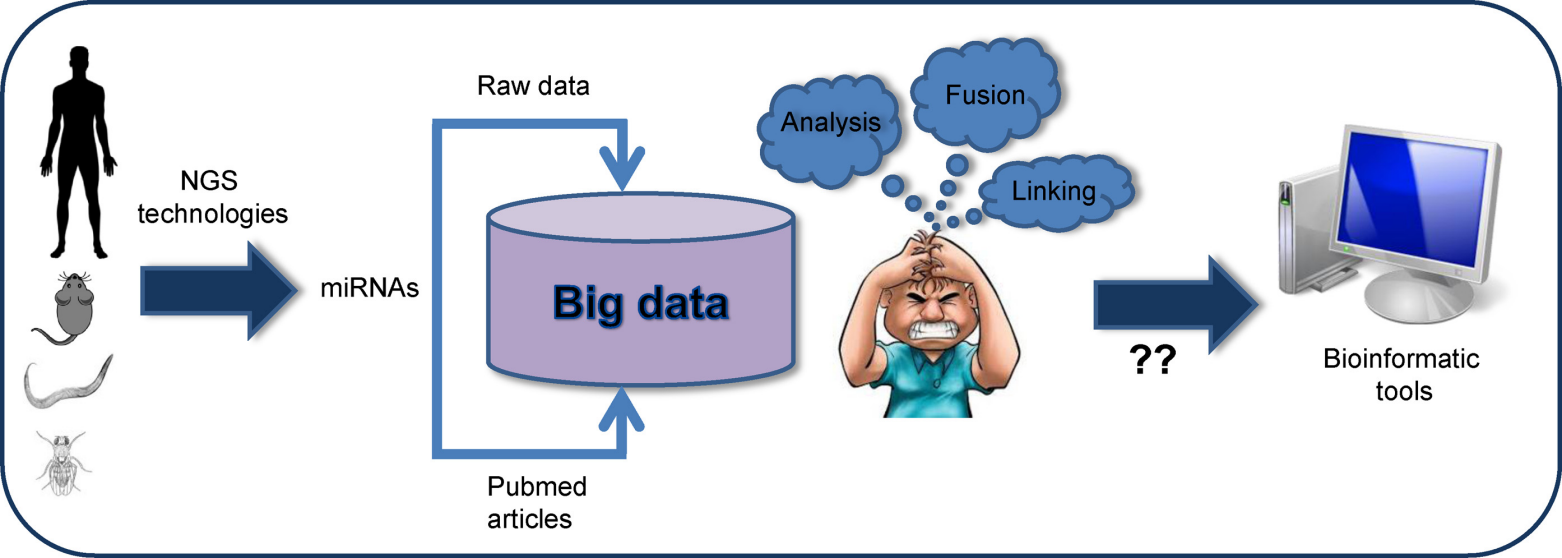
Figure illustrates the complexity of large data sets and the need for bioinformatic tools.

MiRNAs affect nearly all types of cellular pathways, from development to oncogenesis (11). Clearly current miRNA research is not limited to their biogenesis and function. Their clinical implications are now a very topical research issue, because they have been hypothesized to be diagnostic and prognostic biomarkers and therapeutic targets for different human diseases including cancer (12,13). Given their involvement in gene regulation as well as disease processes, experiments have increased at a super-linear rate, generating an exponential flow of data scattered in thousands of articles (Figure 1 illustrates the complexity of large data sets). A large number of bioinformatic tools are now available to manage the mounting data flow. Both basic and applied miRNA research is being enhanced by computational tools and databases. Most applications are accessible through an online interface; researchers around the world can use these cutting-edge analysis pipelines and databases, and even laboratories with poor computational infrastructure can participate in this topical research effort through free online interfaces. We present an overview of the major classes of miRNA tools and databases and discuss critical issues related to their selection.

## BIOGENESIS, FUNCTION AND THERAPEUTIC IMPLICATIONS OF MIRNAS

**Figure 2.**
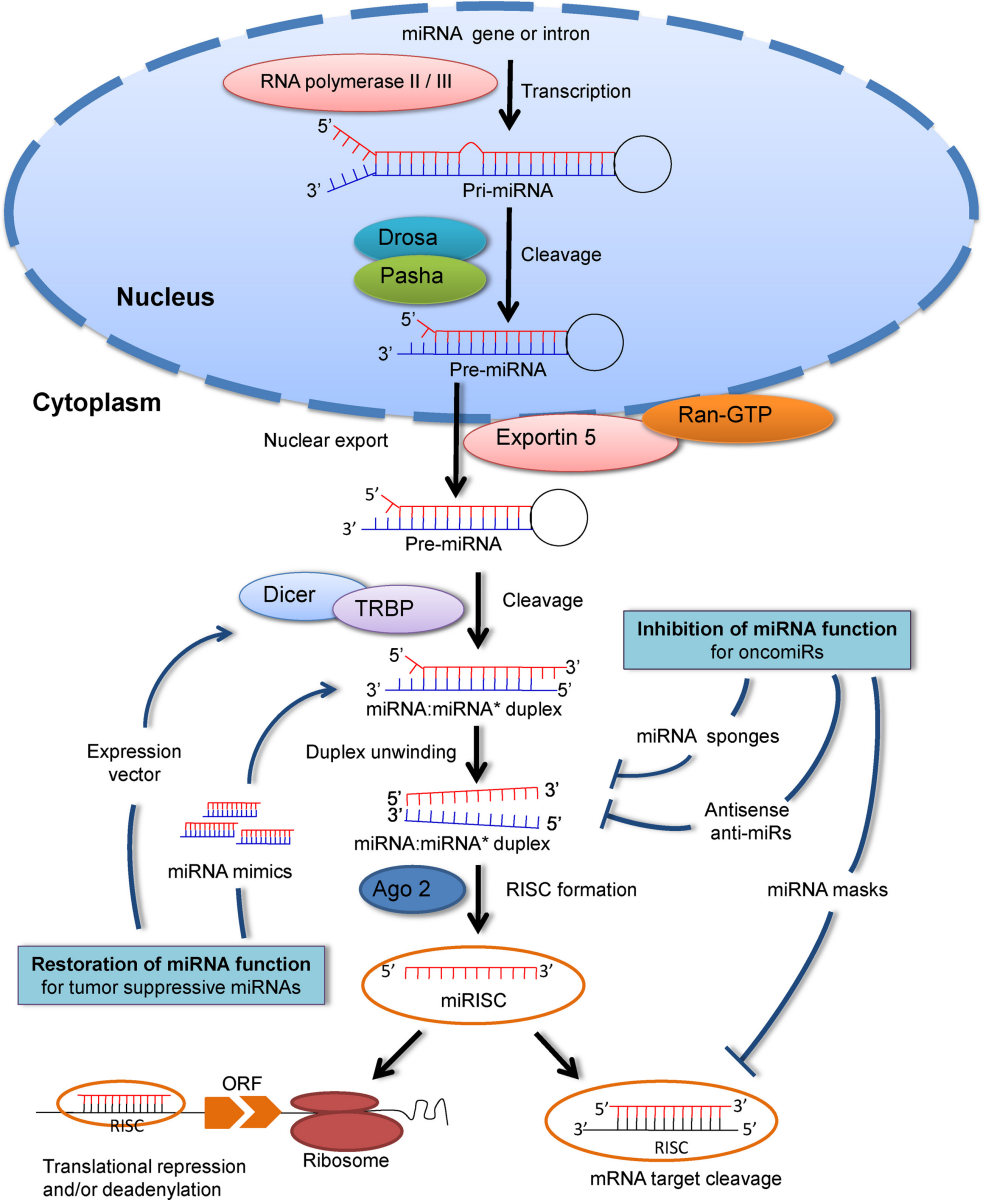
Biogenesis and clinical implications of microRNAs (miRNAs). MiRNA genes are typically transcribed by RNA polymerase II and III and produce primary miRNA (pri-miRNA). Next pri-miRNA is processed to precursor miRNA (pre-miRNA) hairpin structure in the nucleus by the Drosha/Pasha complex and transported into the cytoplasm by Exportin 5. Pre-miRNA is further processed by Dicer-TRBP (TAR RNA binding protein) into a miRNA:miRNA* duplex. After being separated, the mature miRNA loaded into the Argonaute 2 (Ago 2) containing RNA-induced silencing complexes (RISCs). Once the miRISC is assembled, the miRNA drives it to silence target mRNA *via* mRNA cleavage, translational repression or deadenylation. At present two strategies are used for miRNA-based therapeutics in the management of cancer: (i) inhibition of miRNA function for oncomiRs includes miRNA sponges, antisense antimiRs and miRNA masks, (ii) Restoration of miRNA function for tumor suppressive miRNAs includes miRNA mimics and expression vectors.

The biogenesis of miRNA initiates in the nucleus (Figure 2) where it is transcribed into primary miRNA (pri-miRNA) by RNA polymerase II and III (4,14). Transcription of miRNA genes yields long primary transcripts (pri-miRNAs) with a local foldback structure. Next pri-miRNA is cleaved into precursor miRNA (pre-miRNA) by the nuclear microprocessor complex formed by the RNase III enzyme Drosha and its co-factor DiGeorge Syndrome Critical Region 8 (or Pasha) (3). The resulting pre-miRNA hairpin is then exported from the nucleus to cytoplasm by a complex formed by Exportin 5 and Ran-GTP (15,16). In the cytoplasm, the RNase III enzyme Dicer in complex with TAR RNA binding protein (TRBP) cleaves the pre-miRNA hairpin to its mature length (∼21-nt long), giving rise to a miRNA:miRNA* duplex (17). The duplex is then separated and the mature miRNA is loaded together with Argonaute (Ago 2) proteins onto RNA-induced silencing complex (RISC) (3,18). Once the miRISC is assembled, the miRNA drives it to silence target mRNA *via* mRNA cleavage, translational repression or deadenylation (18).

MiRNAs may have a negative or a positive regulatory effect (19). In humans, they usually bind with partial complementarity to 3′UTR regulatory elements on mRNAs called ‘seed sequences’, or to miRNA response elements (MREs) that causes translational repression (20). A major silencing mechanism of miRNAs in animals results in target mRNA destabilization through a cleavage-independent process, affecting transcript level (21,22). A small number of miRNAs also show decoy activity by binding directly to proteins such as RNA-binding proteins, inhibiting interaction with their target RNAs (23). In some cases miRNAs also regulate gene expression at the transcriptional level (24) by binding directly to DNA regulatory elements. In certain cases and cell types they can enhance translation (25).

MiRNAs are frequently deregulated in a wide range of human diseases (26–30), but their involvement in cancer is especially interesting. Numerous studies have examined their function in cancer pathogenesis, diagnosis, prognosis and treatment (31,32). Overexpression or lack of expression of particular miRNAs has been reported to correlate with clinically aggressive or metastatic phenotypes (33,34). A number of miRNAs are tumor-suppressive or oncogenic, in nature according to how they affect cancer cell proliferation (35), and even the same miRNA species can be oncogenic or tumor-suppressive in different tissues (36). The link between cancer and miRNAs was first documented in chronic lymphocytic leukemia, where miR-15 and miR-16 were down-regulated or suppressed (37). Let-7 underexpression was found to be significantly associated with a shorter postoperative survival in human lung cancer independently of disease stage (38). MiRNA upregulation can promote oncogenesis, for instance miR-21 is one of the miRNAs that are most commonly up-regulated in tumor cells, promoting cell proliferation, invasion and migration of cancer cell populations (39,40). Interestingly, miR-221 acts as a tumor suppressor by silencing the KIT oncogene in erythroblastic leukemia (41), but it is overexpressed in liver cancer, where it promotes oncogenesis by targeting tumor suppressor PTEN (42). A growing understanding of the functions of miRNAs is providing insights into the molecular basis of cancers and inspiring research into their use as new biomarkers for cancer diagnosis. Stable extracellular circulating miRNAs, first found in human serum, are another clinically important discovery (43) that suggests the possibility of using miRNAs as non-invasive cancer biomarkers (44–46).

The finding that miRNAs target multiple protein-coding genes and their aberrant perturbations in diverse cancers makes them promising novel therapeutic targets and intervention tools. Perhaps, the most fascinating goal is to use them directly to develop therapeutic strategies for different diseases. Several research efforts currently under way are focusing on developing miRNA therapeutics to treat a wide range of human diseases (Figure 2). The fact that mature miRNA sequences are tiny and frequently conserved across multiple vertebrate species makes miRNAs comparatively easy to target therapeutically (47). Two different approaches are being used to modulate miRNA activity, (i) restoration of its tumor-suppression function by replacing lost miRNA with synthetic miRNA-like RNA duplexes called miRNA mimics or with miRNAs encoded in expression vectors (30,48,49) and (ii) inhibition of miRNA function through chemically modified antimiR oligonucleotides. Since cancer-related miRNAs (oncomiRs) are often overexpressed in various neoplasms, their inhibition would restore the function of their tumor-suppressive target genes. Several miRNA inhibitory agents have been tested in preclinical and clinical studies; they include antisense antimiR oligonucleotides (30), locked nucleic acid antimiRs (50), miRNA sponges (51), miRNA masks (52) and small-molecule miRNA inhibitors (53).

## OVERVIEW OF CURRENT BIOINFORMATIC APPROACHES USED IN miRNA RESEARCH

**Figure 3.**
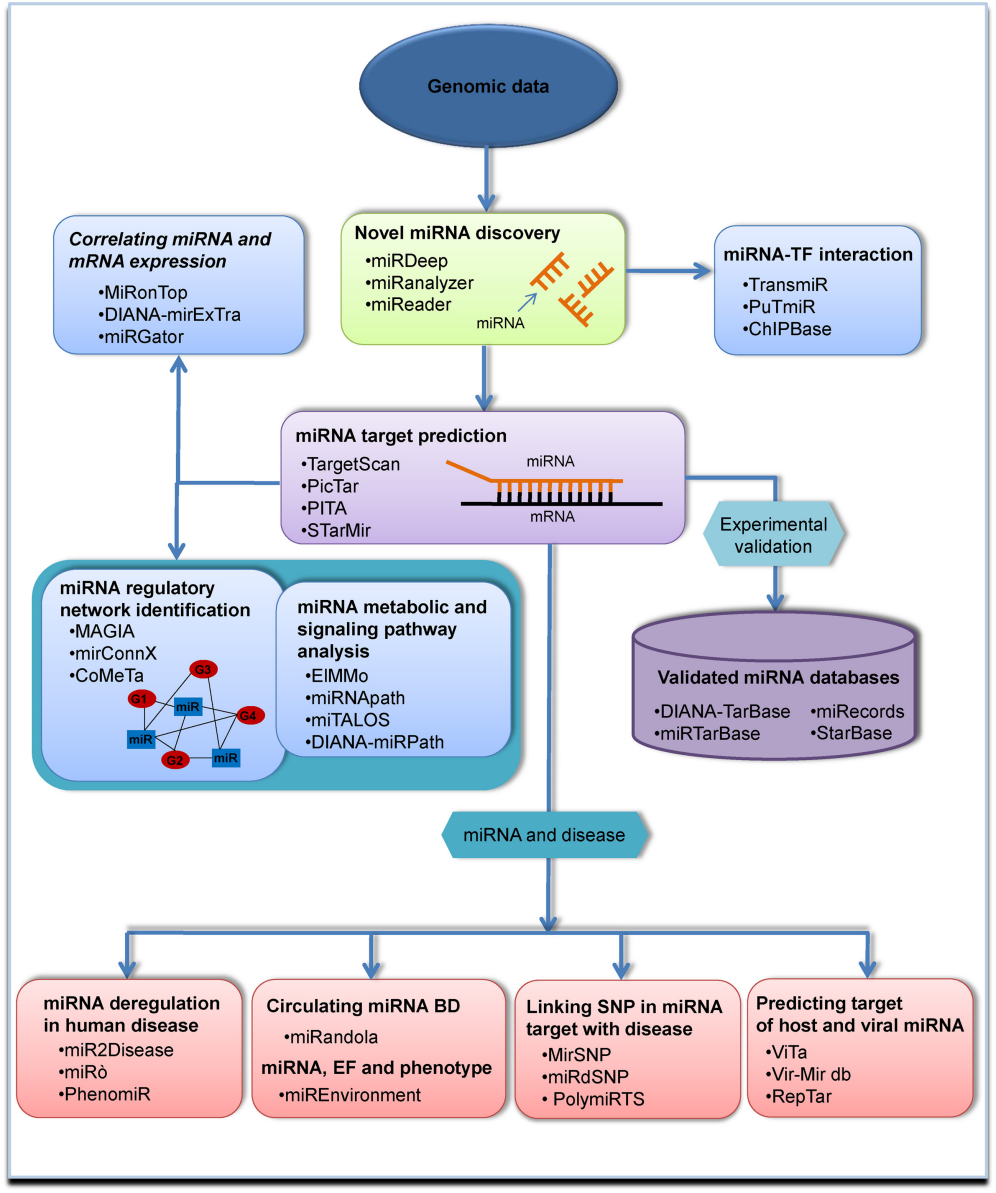
Schematic overview of currently available bioinformatic tools classified according to the main purpose for which they are being used. Sample tools are presented for each category.

In recent years, several bioinformatic tools have been developed to manage the mounting flow of miRNA-related data. Since most contain heterogeneous information, they are difficult to categorize, but in this brief overview we have classified the currently available tools by the main purpose for which they are being used, which include miRNA finding, miRNA target prediction, validated miRNA finding, miRNA expression analysis, identification of miRNA regulatory networks, analysis of miRNA metabolic and signaling pathways, investigation of miRNA and transcription factor (TF) interplay and linking miRNAs to diseases (Figure 3).

### MiRNA discovery

MiRNA identification is complicated and requires an interdisciplinary strategy. Recent technological advances like high-throughput sequencing have made it easier to detect their expression patterns (54). In recent years, biological and bioinformatic approaches have enabled discovery of thousands of miRNAs in plants, animals, unicellular eukaryotes (55) and viruses (56). They are now collected in the **miRBase**, the main online repository of miRNA sequences and annotation. The current miRBase (http://www.mirbase.org) release contains 24 521 miRNA loci from 206 species (30 424 mature miRNA products), including 1872 human miRNA precursors that produce 2578 mature miRNAs (57). The conventional techniques used to discover miRNAs include cloning (58), Northern blotting (59), microarray (60) and *in situ* hybridization (61), which are time-consuming and less cost effective (62). Next generation sequencing (NGS) technology is a reliable and sensitive method to quantify known miRNAs and detect less common ones (63). A variety of algorithms are applied to discover new miRNAs from NGS data. Computational algorithms have been adapted to harmonize experimental approaches directed at identifying and validating new miRNAs. These tools consider some major miRNA features, like sequence conservation among species, and structural features like hairpin and minimal folding free energy (64). Several algorithms have been used to obtain putative secondary structure based on minimum free energy, like RNAfold and Mfold. The major computational tools used for gene finding are described below and listed in Table 1.

**Table 1.** Some selected tools for microRNA discovery

Category	Tool	Machine learning	Type	Applied organism(s)	Input Data^I^ /Training parameters^T^	MRV	Performance			LU	URL	References
							se (%)	sp (%)	a (%)			
Comparative methods	MiRscan	-	Web server	w	Hairpin sequence^I^	-	50	70		2003^N^	http://genes.mit.edu/mirscan/	(65)
	miRseeker	-	Computational method	f	Homologous sequences^I^	-	75	-		2003^N^	http://www.fruitfly.org/seq_tools/miRseeker.html	(66)
Machine learning	ProMir II	HMM	Web server	h, m, r, cn, f, w	Candidate sequence^I^	7, 8	73	96		2006^N^	http://cbit.snu.ac.kr/∼ProMiR2/	(72,73)
	MiRRim	HMM	Method	h,m,r,d	Conserved miRNAs and their surrounding regions^T^	8.2	-	-		2007^N^	http://www.ncrna.org/software/miRRim	(74)
	HHMMiR	HMM	Software without SC	m,cn,z,w,f	miRNA precursors, hairpins sequence^T^	-	84	88		2009^N^	http://www.benoslab.pitt.edu/kadriAPBC2009.html	(75)
	SSCprofiler	HMM	Web server	h	sequence, structure and conservation of miRNAs^T^	12	88.95	84.16		2009^N^	http://mirna.imbb.forth.gr/SSCprofiler.html	(76)
	MiRFinder	SVM	Software with SC	h	Pre-miRNA sequences^T^	8.2	-	-	99.6	2007^N^	http://www.bioinformatics.org/mirfinder/	(78)
	BayesMiRNA Find	NBC	Computational method	m	Genomic sequence^I^	-	97	91		2006^N^	http://wotan.wistar.upenn.edu/miRNA	(79)
	MatureBayes	NBC	Web tool with SC	h,m,f,z	Sequence and secondary structures of pre-miRNA^T^	14	-	-	80	2010^N^	http://mirna.imbb.forth.gr/MatureBayes.html	(80)
NGS based	miRDeep/ miRDeep2	-	Software with SC	h,m,d,w,f, ss,pl,sa	Deep sequencing data^I^	10, 16	89	-	98.6–99.9	2012^Y^	https://www.mdc-berlin.de/en/research/research_teams/systems_biology_of_gene_regulatory _elements/projects/miRDeep	(81,82)
	miRanalyzer	-	Web server and stand-alone tool	h,m,w,f,z + 33 more	Read-count files and multi-fasta files of small-RNA seq data^I^	12,16, 20	-	-		2013^Y^	http://bioinfo2.ugr.es/miRanalyzer/miRanalyzer.php	(83,84)
	miReader	-	Software without SC	h,w,f,z	Small-RNA seq read data^I^	19	94.05	94.7		2013^Y^	http://scbb.ihbt.res.in/2810--12/miReader.php	(85)

Machine learning: HMM, hidden Markov model; SVM, support vector machine; NBC, Naive Bayes classifier

SC, Source code

Applied organisms: h, human; m, mouse; r, rat; f, fly; w,worm; cn, chicken; d, dog; z, zebrafish; ss, sea squirts; pl, planaria; sa, sea anemone

MRV, MiRBase Realese Version

Performance: sensitivity (se), specificity (sp) and accuracy (a) as found in the related articles. LU, last updated; ^Y^if updated over the past 5 years, ^N^not updated

Early bioinformatic methods predicted putative miRNAs in genome sequences by targeting secondary RNA structure, i.e. conserved hairpin structures that are characteristic of miRNA precursor sequences in related species. **MiRscan** (65) and **miRseeker** (66) are the main tools that target conserved intragenic sequences that can form hairpin structures based on RNAfold and Mfold, respectively. MiRscan then compares the identified structures with known miRNA features like 3′ and 5′-stem conservation, whereas miRseeker selects hairpins sharing similar nucleotide divergence patterns to the reference set. MiRscan and miRseeker were first applied to identify miRNAs in nematodes and flies, respectively, and a large number of predicted candidates were then validated experimentally.

However, since tools based on comparative methods essentially focus on evolutionarily conserved miRNAs, they are limited to discovery of novel miRNAs. Machine-learning methods have subsequently been devised to predict novel miRNAs. These techniques have improved the prediction of unknown miRNAs by extending the analysis beyond sequence and structural properties. Machine-learning algorithms allow computer programs to ‘learn’ from the information collected from previously verified miRNAs, used like positive miRNA standards. Algorithms include hidden Markov model (HMM), Naïve Bayes classifier (NBC) and support vector machine (SVM) (62). HMMs offer pattern recognition among data sets, in particular nucleotide sequences (67). Naïve Bayes is a classification model that is obtained by applying a relatively simple method to a training data set. NBC calculates the probability that an example belongs to a certain class (68,69). SVM is a classifier that categorizes objects based on a set of features for each object. It compares vectors from a positive and a negative class and provides a hyperplane producing the best separation margin between them (70,71). Several tools based on these approaches have been developed to predict miRNAs from different species. For instance, the HMM-based tool **ProMir** (72) is a probabilistic co-learning model based on conserved sequences and secondary structures that is applied to predict human miRNA genes. The improved version, ProMiR II (73), provides additional filtering criteria such as G/C ratio, conservation score, entropy and free energy of candidate sequences. Prediction of conserved and non-conserved miRNA genes is also possible by adjusting the filtering criteria. Use of appropriate training data sets allows application to all species. **MiRRim** (74) is another HMM-based tool that considers both evolutionary and secondary structure features of miRNA genes to achieve high-performance identification of new human miRNAs, in particular those clustering with known miRNAs. **HHMMiR** (75), predicts *de novo* miRNA hairpins in the absence of evolutionary conservation. The method implements a hierarchical HMM that utilizes region-based structural as well as sequence information of miRNA precursors. Another freely available prediction tool, **SSCprofiler** (76,77), harnesses a probabilistic method based on *Profile* HMMs, trained to recognize key biological miRNA features such as sequence, structure and conservation, to identify novel miRNA precursors. This trained classifier is applied to identify novel miRNA gene candidates located in cancer-associated genome regions.

**MiRFinder** (78) is an SVM-based tool that compares genome-wide and pair-wise sequences between related species. It identifies hairpin structures from a set of miRNA candidates and excludes non-robust structures by SVM analysis of 18 different parameters. Pair-wise genome alignments have shown that it can be used for genome-wide pre-miRNA predictions; however, it may fail to detect species-specific pre-miRNAs. The NBC-based program **BayesmiRNAfind** (79) is a computational approach that predicts known miRNAs based on their secondary structure and sequence for a specific genome as the input. Another NBC-based computational tool, **MatureBayes** (80), identifies mature miRNA candidates based on sequence and secondary structure information of their miRNA precursors. The method predicts the start position of experimentally verified, mature, human and mouse miRNAs. It considers both positive (true mature miRNAs) and negative (same-size non-mature miRNA sequences) examples to optimize sensitivity and specificity. It is significantly more accurate than ProMiR and BayesMiRNAfind (80). A list of miRNAs discovered *via* traditional evolutionary conservation approaches and machine learning-based techniques is provided as Supporting Material S1.

Several tools have been devised to predict miRNAs from NGS data. The most common are **miRDeep/miRDeep2** (81,82) and **miRanalyzer** (83,84). Both can find previously known and novel miRNAs. Another recent tool, **miReader** (85), identifies mature miRNAs directly from NGS read data without the need for genomic sequences or homologous references. Experimental techniques such as molecular cloning, sequencing or hybridization are typically used to validate predictions (86).

Establishing the biological function of the novel miRNAs discovered with these tools requires additional and more sophisticated bioinformatic analysis. Most online bioinformatic resources take into consideration only known miRNAs. However, a number of tools described in the next section, such as TargetScan v5.2 (*via* the link ‘Targetscan custom’) (87), MiRDB (*via* the link ‘custom prediction’) (88), DIANA-microT v3.0 (*via* the link ‘predict for your microRNA sequence’) (89) and RNAhybrid (90), include a functionality that can be applied to identify the targets of novel miRNAs. Advanced users can download the TargetScan code and run the TargetScan algorithm on any set of seed regions of interest. Miranda (91) and RNAhybrid can also be downloaded and run locally to identify the targets of user-provided miRNA sequences.

The studies that have employed these tools for miRNA finding are listed in Supporting Material S2; additional miRNA finding tools are reported in Supporting Material S3.

### MiRNA target prediction

It is well established that miRNAs down-regulate gene expression by targeting 3′UTRs of mRNAs through sequence-specific binding. Knowing miRNA targets is essential to understand their function. A single miRNA can target multiple genes and several miRNAs can target a single gene (92). Since more than one-third of human genes appear to have been under selective pressure to maintain their pairing to miRNA seeds, miRNAs are clearly involved in a broad range of cellular processes (87). Unlike plant miRNAs, which usually bind to their targets with perfect complementarity (93), animal miRNAs have limited complementarity which makes it more difficult to determine possible miRNA targets with high specificity (20). Seed regions (nucleotides 2–7 in the 5′ region of miRNAs) are considered crucial for mRNA targeting. Most of the available algorithms require Watson–Crick pairing with the targeted site (94). Algorithms depending on simple base-pairing rules result in high false positive rates (94). Target prediction algorithms are evolving in parallel with the growing understanding of miRNAs. Validating a possible miRNA target in the laboratory is expensive and time-consuming, since each miRNA has a large number of potential target sites. Computational approaches help reduce their number for experimental validation.

In most tools prediction thresholds can be entered, to manage prediction sensitivity or accuracy level. The algorithms take into account several features to increase prediction efficiency, including (i) seed complementarity between miRNA and mRNA strands; (ii) evolutionary conservation of miRNA target sites among species; (iii) free energy of the miRNA:mRNA duplex; (iv) target site accessibility; and (v) the contribution of multiple binding sites (95).

A number of online computational tools have been developed to predict putative miRNA targets. Although they have been extensively reviewed (94,96,97), a brief description of some of those used most commonly is reported below for the sake of completeness (see Table 2 for their general features).

**Table 2.** Some selected bioinformatic resources for microRNA (miRNA) target prediction

Group	Tools	Type	Organism(s)	Site coverage on mRNA				Input data	MRV	PER	USD	UA	UL	NTI	LU	URL	References
				P	5′ UTR	CDS	3′ UTR										
Single platform	TargetScan	Web server^A^	h, m, r, d, cn, c, rh, cw, o, fr, z, f, w				√	GS, MF	10.1, 17,21	69% sp^a^	Y	N	A	-	2015 ^Y^	http://www.targetscan.org/	(10,87,98)
	RNAhybrid	Web server /software^A^	f, w				√	TS, MS	-	58% se^b^	Y	Y	Ad	-	2006^N^	http://bibiserv.techfak.uni-bielefeld.de/rnahybrid/	(90,100)
	PicTar	Web server^NA^	h, m, f, w				√	MI, GI	6	70% sp^a^	N	N	A	-	2007^N^	http://pictar.mdc-berlin.de/	(99)
	rna22	Web server^NA^	h, m, f, w		√	√	√	MS, TS, RT, MD	16, 18, 19, 21	81% sp^a^	Y	N	I	-	2015^Y^	https://cm.jefferson.edu/rna22/	(101,102)
	PITA	Web server /software^A^	h, m, f, w				√	MI, GI, US, MS	11	0.76 AUC score	Y	Y	A	-	2008^N^	http://genie.weizmann.ac.il/pubs/mir07/	(103)
	miRDB	Database/web server^NA^	h, m, r, d, cn		√	√	√	MI, GI, GA, GS, MS, TS	9.1, 10, 13, 18, 21	-	Y	Y	A	-	2015^Y^	http://mirdb.org/	(88,104)
	microRNA.org/Miranda	Database /software^A^	h, m, r, f, w				√	MI, GS	10, 11, 15	76% sp^a^	Y	N	A	-	2010^Y^	http://www.microrna.org/	(105,184)
	DIANA-microT-CDS	Web server/software^A^	h, m, f, w			√	√	MI, GI, KD	18	65% se	Y	N	A	-	2013^Y^	http://www.microrna.gr/webServer	(107)
	STarMir	Web server^NA^	h, m, w		√	√	√	MI, MS, GI, TS	20	-	Y	N	A	-	2014^Y^	http://sfold.wadsworth.org/cgi-bin/starmirtest2.pl	(109)
Integrated platform	miRNAMap	Database^NA^	h, m, r, d, cn, o, fr, z, f, w, p, mq				√	MI, GI	6, 9.2		N	Y	A	3	2008^N^	http://mirnamap.mbc.nctu.edu.tw/	(185,186)
	MiRror/miRror Suite	Database^NA^	h, m, r, f, w, z		√	√	√	MI, GI	-		Y	Y	A	15	2014^Y^	http://www.proto.cs.huji.ac.il/mirror/index.php	(187,188)
	miRTar	Web server^NA^	h		√	√	√	MA, MS, GI,GS, PN	15		Y	Y	I	4	2011^Y^	http://mirtar.mbc.nctu.edu.tw/human/	(189)
	miRWalk	Database^NA^	h, m, r	√	√	√	√	GS, GI, MI,MA, PN,CT, OT, MT	20		Y	Y	A	8	2015^Y^	http://www.umm.uni-heidelberg.de/apps/zmf/mirwalk/	(190)
	mirDIP	Web server^NA^	-		√	√	√	GS, MI	-		Y	Y	A	12	2012^Y^	http://ophid.utoronto.ca/mirDIP/	(95)
	ComiR	Web server^A^	h, m, f, w				√	MI, TS	-		Y	Y	A	4	2014^Y^	http://www.benoslab.pitt.edu/comir/	(191)
	mirTarPri	Web server^NA^	h					PD, MI	-		N	N	A	6	2013^Y^	http://210.46.85.180:8080/mirTarPri/	(192)
	miRmap	Web server/software^A^	h, m, r, cw, o, cn, c, z				√	MI, GS, TS	19		Y	Y	Ad	4	2013^Y^	http://mirmap.ezlab.org/	(193,194)
	ToppMiR	Web server^NA^	h					MI, GS, GI	-		N	Y	Ad	7	2014^Y^	https://toppmir.cchmc.org/	(195)

Type: ^A^source code available; ^NA^not available

Organisms: h, human; m, mouse; r, rat; d, dog; cn, chicken; c, chimpanzee; rh, rhesus; cw, cow; o, opossum; fr, frog; z, zebrafish; f, fly; w, worm; p, pufferfish; mq, mosquito

Input data: GS, gene symbol; MF, miRNA family; TS, target sequence; MS, miRNA sequence; RT, RNA type; MD, miRNA database; GA, GeneBank accecetion; MI, miRNA ID; GI, Gene ID; US, UTR sequence; KD, kegg descriptions, MA, miRBase accession number, PN, pathway name; CT, chromosome targets; OT, OMIM targets; MT, mitochondrial targets; PD, prediction database

MRV: miRBase release version

PER, performance: se, sensitivity; sp, specificity; AUC, area under the curve; ^a^ Ahmadi *et al*. (196), 2013; ^b^Zhang *et al*. (197)

USD, user submitted data: Yes (Y) or No (N); UA, user adjustability: Yes (Y) or No (N); UL, user level: all (A), advanced (ad), I (intermediate); NTI, no. of tools integrated; LU, last updated: ^Y^if updated over the past 5 years, ^N^not updated.

**TargetScan** (10,87,98) is a web tool that predicts miRNA targets by searching for conserved and non-conserved sites. It detects targets in the 3′UTR of protein-coding transcripts by base-pairing rules (seed matching); it also predicts secondary structure to calculate the free energy of predicted duplexes. Several features like 3′ compensatory pairing, local AU content and position contribution make up the score. **PicTar** (99) identifies binding sites for a single miRNA and multiple sites regulated by different miRNAs acting co-operatively. It uses a pair-wise alignment algorithm to find conserved sites across multiple species. To enhance prediction it also considers miRNA clustering and co-expression together with ontological information, such as miRNA time and tissue specificity and their potential targets. **RNAhybrid** (90,100) predicts multiple potential miRNA binding sites in large target RNA**s** and considers the free energy of miRNA:mRNA duplexes. In contrast, **rna22** (101,102) is a pattern-based approach to find miRNA binding sites and corresponding miRNA:mRNA complexes without a cross-species sequence conservation filter. It can identify putative miRNA binding sites even though the targeting miRNA is unknown.

The major miRNA target prediction feature of **PITA** (103) is target site accessibility. This is a parameter-free model for miRNA-target interaction that computes the difference between the free energy gained from miRNA-target duplex formation and the energy cost of unpairing the target to make it accessible to the miRNA. **MiRDB** (88,104) is an online database for miRNA target prediction and functional annotations with a focus on mature miRNAs. It provides a web interface for target prediction generated by an SVM machine learning algorithm and has a wiki editing interface for interactive community-annotated miRNA functional catalog. The web interface **microRNA.org** predicts candidate targets using the miRanda algorithm and scores them with the mirSVR machine learning method for their potency to repress targeted genes (105). MiRanda recognizes target sites using features like sequence complementarity between mature miRNAs and the free energy of the duplex (91).

**DIANA-microT-CDS** (89,106,107) is the latest version of DIANA-microT, an algorithm that incorporates a machine-learning approach to identify the most relevant features extracted from photoactivatable-ribonucleoside-enhanced cross-linking immunoprecipitation (PAR-CLIP) data (108). This enables the algorithm to learn the features associated with miRNAs whose binding site is known both in coding sequences (CDs) and 3′UTRs. For target prediction it considers features like binding category weight, distance to the nearest end of the region (CDS or 3′UTR) or to an adjacent binding site, the predicted free energy of the duplex, conservation and AU content. Though DIANA-microT is a web-based tool, advanced users are offered a Taverna plugin that provides additional options and a non-web interface. Another tool, **STarMir** (109), implements logistic prediction models developed with miRNA binding data from CLIP studies (110). To predict miRNA binding sites, STarMir computes comprehensive sequence, thermodynamic and target structure features and a logistic probability as a measure of confidence for each predicted site (3′ UTR, CDS and 5′UTR). Several research groups have used these tools for target prediction (the relevant studies are listed in Supporting Material S2). Additional and freely available online tools for miRNA target prediction are presented in Supporting Material S3.

The information reviewed above clearly indicates that current target prediction platforms are based on different prediction assumptions and models, a fact that has considerably hampered the selection by researchers of the appropriate tool for their specific requirements. Therefore, checking the underlying assumptions, strengths and limitations of a target prediction tool before employing it would be a practical approach. Combining results from multiple tools is a common, often encouraged practice to minimize false positive and/or negative outputs. Even though all the tools reviewed above have predictive power, they all have limitations related to the features incorporated into them. Thus, a tool relying exclusively on seed match for miRNA target identification is unlikely to show whether the target site sequence is evolutionarily conserved or accessible for binding and calculate the energy required for miRNA:mRNA duplex formation. There is evidence that many non-conserved binding sites in 3′UTRs are functional (111). Therefore, exclusive use of conservation-based miRNA target prediction systems is unlikely to capture such miRNA:mRNA interactions.

Not all miRNA target prediction tools are consistently updated. Regular updating is important, since miRNA nomenclature changes continuously and novel miRNAs are added to the miRBase every year (the list provided in Supporting Material S4 reports the year of release of each miRBase version). In a recent miRBase version, v21, 278 ‘High-Confidence’ human miRNAs were identified based on structural analysis of precursor miRNAs combined with expression counts (57). RNAhybrid uses an older version of the web server and has not been updated recently; the facts that it does not offer default values and requires adjustment of complex settings with user-supplied input make it difficult to use for beginners. The web servers of Pictar and PITA are more than 5 years out of date. However, PITA offers a downloadable version compatible with user-provided data. MiRanda, another widely used but dated algorithm, is also downloadable. Other tools such as TargetScan, rna22, STarMir, DIANA-microT-CDS and miRDB, are regularly updated. TargetScan and miRDB are those most frequently updated, and use the High-Confidence status of a miRNA for functional miRNA identification.

There is evidence that miRNA-binding sites within coding sequences are also involved in controlling gene expression (112). Tools that predict only the target in conserved 3′UTRs are unable to predict miRNA:mRNA interactions in other regions. Among the tools reviewed above DIANA-microT-CDS can identify miRNA targets in CDSs as well as 3′UTRs, and rna22, miRDB, and STarMir can do so in CDSs, 5′UTRs and 3′UTRs.

Even though these miRNA target prediction approaches can be used independently, some web-based integrated platforms have been built in recent years to combine multiple algorithms (Table 2). The results they generate cannot be used directly, but require experimental validation. However, evaluation of these tools is beyond the scope of the present review.

### Finding validated miRNA information

Potential miRNAs obtained even from the most efficient prediction tools require experimental validation. This can be accomplished with several approaches. Well-established techniques for gene-specific experimental validation include qRT–PCR, luciferase reporter assays and western blotting. High-throughput sequencing (HITS) techniques are also available such as microarrays, proteomics, and sequencing-based methodologies such as RNA-Seq, HITS-CLIP, PAR-CLIP and Degradome-Seq (92). Some experimentally validated miRNA target databases that collect, curate and/or analyze the relevant literature are now available (Table 3). The studies that have used them are reported in Supporting Material S2.

**Table 3.** Bioinformatic resources to deal with different aspects of microRNA related research

Category	Tools	Type	Organism(s)	Input data	MRV	LU	URL	References
Finding validated miRNA information	DIANA-TarBase	Database	h,m,r,f,w,z + 18 more	MI	21	2015^Y^	http://www.microrna.gr/tarbase	(113,114)
	miRTarBase	Database	h,m,r,f,w,z + 12 more	MI, MF, GS,KP,VM, DN,PIMD, ML,GL	20	2013^Y^	http://mirtarbase.mbc.nctu.edu.tw	(115,116)
	miRecords	Database	h,m,r,f,w,z,cn,sh	MI,GI	11,20	2013^Y^	http://c1.accurascience.com/miRecords/	(117)
	StarBase	Database	h,m,w + 3 more	MI,GS	15, 20	2014^Y^	http://starbase.sysu.edu.cn/	(118,119)
Correlating miRNA and mRNA expression	MiRonTop	Database	-		15	2010^N^	http://www.microarray.fr:8080/miRonTop/index	(120)
	DIANA-mirExTra	Web server	h,m	GL	10	2010^N^	http://diana.cslab.ece.ntua.gr/hexamers/	(121)
	mESAdb	Database	h,m,z	MI.ML	15	2010^N^	http://konulab.fen.bilkent.edu.tr/mirna/mESAdb_information.php	(123)
	miRGator	Database	h	MI,	18	2013^Y^	http://mirgator.kobic.re.kr/index.html	(127)
miRNA regulatory network identification	MAGIA	Web server	h	ML, GI, GED,MED	14	2009^N^	http://gencomp.bio.unipd.it/magia/start/	(128)
	mirConnX	Web server	h, m	GED,MED	14	2011^Y^	http://www.benoslab.pitt.edu/mirconnx	(130)
	CoMeTa	Database	h	GB,KP,MI	13	2012^Y^	http://cometa.tigem.it/	(131)
miRNA metabolic and signaling pathway analysis	ElMMo	Web server	h,m,r,w,f,z	MRI, MRL, MI	11	2009^N^	http://www.mirz.unibas.ch/ElMMo/	(135)
	miRNApath	Database	h,m,r,d	GL,MI	9.2	2007^N^	http://lgmb.fmrp.usp.br/mirnapath	(136)
	miTALOS	Web server	h, m	MI, MC,TT, KP, NP	-	2011^Y^	http://hmgu.de/cmb/mitalos	(137)
	miRSystem	Database	h, m	ML, PD, GS	20	2015^Y^	http://mirsystem.cgm.ntu.edu.tw/	(138)
	DIANA-miRPath	Web server	h, m, r, f,w,z cn,	MI	18, 21	2015^Y^	http://snf-515788.vm.okeanos.grnet.gr/dianauniverse/index.php?r=mirpath	(122,139,140)
miRNA and transcription factor interaction	TransmiR	Database	h,m,r,w + 12 more	TF, MI	-	2013^Y^	http://cmbi.bjmu.edu.cn/transmir	(142)
	PuTmiR	Database	h	MI	14	2010^N^	http://www.isical.ac.in/∼bioinfo_miu/TF-miRNA/TF-miRNA.html	(143)
	CircuitsDB	Database	h,m	TF, MI,GS	9.2	2010^N^	http://biocluster.di.unito.it/circuits/index.php	(144)
	MIR@NT@N	Software	paper		14	2010^N^	http://maia.uni.lu/mironton.php	(145)
	ChIPBase	Database	h,m,d,cn,f,w	TF, MI, RR	17	2012^Y^	http://deepbase.sysu.edu.cn/chipbase/	(146)
miRNA deregulation in human disease	miR2Disease	Database	h	MI,DN,TG	11	2008^N^	http://www.mir2disease.org/	(151)
	miRò	Database	h,m	MI,GS, DN,P,T	12	2009^N^	http://ferrolab.dmi.unict.it/miro/	(152)
	PhenomiR	Database	h	MI,DN,PI, T/C,SD,M,TG	12	2011^Y^	http://mips.helmholtz-muenchen.de/phenomir/	(153)
	OncomiRDB	Database	h	MI, TT, T, TG,F	16,20	2014^Y^	http://bioinfo.au.tsinghua.edu.cn/member/jgu/oncomirdb/index.php	(154)
	miRCancer	Database	h	MI, Cancer name	18	2015^Y^	http://mircan-cer.ecu.edu/	(155)
	HMDD	Database	h	MI,DN	20	2014^Y^	http://210.73.221.6/hmdd	(156)
Extracellular circulating miRNA	miRandola	Database	h	MI,MF,MT,D, S,PB	18	2015^Y^	http://atlas.dmi.unict.it/mirandola/index.html	(158)
Linking miRNA, environmental factors and phenotype	miREnvironment	Database	h, m, r, d, cn, c, cw, fr, z, p w	MI, EF,P,S	17	2012^Y^	http://210.73.221.6/miren	(165)
Linking polymorphisms in miRNA target with human disease	Patrocles	database	h,m,r,c,cn,d,cw	-	11	2009^N^	http://www.patrocles.org/	(171)
	MicroSNiPer		h,m	US,GI,SI	15,19	2012^Y^	http://epicenter.ie-freiburg.mpg.de/services/microsniper/	(172)
	Mirsnpscore	Database	h	GI,MI,SI	16	2010^N^	http://www.bigr.medisin.ntnu.no/mirsnpscore/	(174)
	MirSNP	Database	h	GN, R,SI,MI, GL, MRL,SL	18	2012^Y^	http://202.38.126.151/hmdd/mirsnp/search/	(175)
	miRdSNP	Database	h	GN,MI, PM,SI,D,DS	18	2011^Y^	http://mirdsnp.ccr.buffalo.edu/	(170)
	PolymiRTS	Database	h,m	SI,MI,GI,GD, T,GO	17,20	2013^Y^	http://compbio.uthsc.edu/miRSNP/	(176,177)
Somatic mutations in miRNAs and their target sites	SomamiR	Database	h	CL, MI, GI, GS	17	2012^Y^	http://compbio.uthsc.edu/SomamiR/	(179)
	miR2GO	Web server	h	MSE, MI, SI, MP	21	2015^Y^	http://compbio.uthsc.edu/miR2GO/home.php	(180)
Prediction of cellular target of host and viral miRNA	ViTa	Database	H,m,r,cn	VI,MI,D,IT	8.2	2006^N^	http://vita.mbc.nctu.edu.tw/	(181)
	Vir-Mir db	Database	h,m,r,z	GB,RA, VN	9	2007^N^	http://alk.ibms.sinica.edu.tw/	(56)
	Bi-Targeting	Method	h	-	14	2010^N^	http://www.cs.bgu.ac.il/∼vaksler/BiTargeting.htm	(182)
	RepTar	Database	h, m	MS, MI, GN	15	2010^N^	http://reptar.ekmd.huji.ac.il/	(183)

Input data: KP, KEGG pathway; VM, validated method; DN, disease name; ML, miRNA list; GL, gene list; GS, Gene symbol; RR, regulatory region;,DN, disease name; TG, target gene, TT, tissue type; T, tumor; F, function; EF, environmental factor; P, phenotype; S, species, US, 3′ UTR sequence; SI,SNP ID; MS, miRNA seed, GN, gene name; MR, mRNA ID; MI, miRNA ID; GL, gene list; MRL, mRNA list; SL, SNP list; DS, distance between SNP and miRNA target site in nucleotides, GD, gene description; T, trait; GA, GO accession; CL, Chromosome Location, VI, virus ID; IT, infected tissue of virus; GB, GenBank Id of virus; RA, RefSeq accession of virus; VN, viral scientific name; MS, miRNA source; GED, gene expression data; MED, miRNA expression data; GB, GO term Biological Process FAT; MRI, mRNA ID; MC, miRNA cluster; NP, NCI pathway; PD, pathway database; MSE, miRNA sequence; MP, miRNA pair

Organisms: h, human; m, mouse; r, rat; d, dog; cn, chicken; c, chimpanzee; cw, cow; fr, frog; z, zebrafish; f, fly; w, worm; p, pig; sh, sheep

MRV: miRBase release version

LU, last updated: ^Y^updated over the past 5 years, ^N^not updated.

**DIANA-TarBase** (113,114) is a manually curated target database. The latest version (v7.0) contains more than half a million miRNA-target interactions (MTIs), curated from published experiments performed with 356 different cell types from 24 species. It indexes 9- to 250-fold more entries than any other relevant database. It incorporates data derived from 154 CLIP-Seq/CLASH data sets as well as more than a hundred other high-throughput data sets. The database enables retrieval of positive and negative experimental results, experimental methodology used, experimental conditions including cell/tissue type and treatment. The data set is freely available for download. The **miRTarBase** (115,116) has accumulated more than 50 000 MTI from 18 species that are collected by manual screening of the relevant literature after data mining of the text, to filter research articles related to functional studies of miRNAs. MTIs are validated experimentally by reporter assay, western blotting, microarray and NGS experiments. The miRTarBase provides an updated collection through comparisons with similar, previously developed databases. **MiRecords** (117) is also a manually curated database hosting 2705 records of interactions between 644 miRNAs and 1901 target genes in 9 animal species. It also contains predicted targets calculated using 11 different algorithms. Another tool, **StarBase** (118,119), is designed for multiple tasks including miRNA:mRNA interaction based on CLIP-Seq data. Among databases containing validated miRNA information, DIANA-TarBase is the most frequently updated, and is associated with the latest miRBase version (v21), which offers High-Confidence miRNA sets. It also supports the largest number of species and entries. Three other databases are linked to miRBase v20. The data sets from all databases are available for free download.

### Correlating miRNA and mRNA expression

Numerous tools provide miRNA and miRNA target prediction. Despite a significant number of studies, our understanding of the molecular mechanisms underlying miRNA targeting is still incomplete. MiRNA functional analysis and expression analysis could help identify potential targets and uncover biologically important relationships. Several online bioinformatic resources for miRNA expression analysis are also available; most combine target prediction with expression data. Some tools that use miRNA expression data are described above (Table 3). The list of studies that have used these tools is reported as Supporting Material S2.

**MiRonTop** (120) is an online Java application that identifies the potential involvement of miRNAs in a given biological system using DNA microarrays or HITS data. It provides fast characterization of the most significant mRNA targets according to several prediction approaches. It also provides options to estimate enrichment scores according to the spatial distribution of predicted target sites along the transcript, since true sites may be preferentially located in the vicinity of stop codons and polyA sites. It provides graphs of miRNA enrichment associated with up- or down-regulated transcripts and summary tables of selected mRNA targets and their functional annotations by Gene Ontology. **DIANA-mirExTra** (121) is a web server that identifies primarily the miRNAs inducing gene deregulation by targeting six nucleotide-long motifs (hexamers) that are overrepresented in the 3′ UTR sequences of deregulated genes. Once miRNAs of interest are detected, the user can directly view their predicted targets as produced by DIANA-microT 3.0 (89). To learn how gene deregulation may contribute to disease development or other processes of interest, an integrated tool, DIANA-mirPath (122), suggests biological pathways in which miRNA targets of interest are more likely to be involved. **mESAdb** (123) is an interactive and expandable analytical tool that uses miRNA sequence and expression data from multiple taxa. MESAdb analysis modules allow (i) mining selected miRNA expression data sets for a list of miRNAs; (ii) pair-wise multivariate analysis of expression data sets within and between taxa; and (iii) association of miRNA lists or of miRNAs with a given motif with annotation databases, HUGE Navigator (124), KEGG (125) and GO (126). The possibility of uploading and analyzing user-specified data sets makes mESAdb an interactive and expandable analysis tool for miRNA sequence and expression data. Finally, **miRGator v3.0** (127) is an integrated portal collecting deep sequencing miRNA data that has become the principal resource on miRNA diversity and expression. It encompasses miRNA diversity, expression profiles, target relationships and various supporting tools. The weakness of these tools is that they are not regularly updated. Only miRGator is linked to miRBase v18, whereas the others are using older versions. Additional miRNA expression analysis tools are presented in Supporting Material S3.

### MiRNA regulatory network identification

Since miRNAs can have multiple targets, and each protein-coding gene can be targeted by multiple miRNAs that make up a complex regulatory network. The investigation of the biological importance of the miRNA-target interaction network is an exciting and challenging task. Construction of networks enables modeling complex biological systems. Since miRNAs play a key role in many processes and pathways, it is crucial to have tools that can integrate miRNA-related data into networks. In this section we describe some tools that combine miRNA-related data to create interaction networks that model and describe the molecular that involve miRNA regulation. Most of these tools also offer computational facilities for the visualization and analysis of such networks. This class of tools offers an interface to deal with network-oriented data (Table 3). The studies that have employed these tools are reported in Supporting Material S2.

**MAGIA** (miRNA and genes integrated analysis) (128) is a web tool for the integrated analysis of target predictions and for miRNA and gene expression data. It offers an interface to construct bipartite regulatory networks of the best putative miRNA:mRNA interactions. The interactive bipartite regulatory network is reported together with the corresponding browsable table of relationships. A hyperlink allows functional enrichment analysis through the **DAVID** tool (129) on the desired number of target genes. The user can further investigate each mRNA, miRNA or miRNA:mRNA interaction and employ it for different queries. Similarly, **mirConnX** (130) is a web interface for inferring, displaying and parsing mRNA and miRNA gene regulatory networks. It combines sequence information with gene expression data analysis to create a disease-specific, genome-wide regulatory network. Another tool **CoMeTa** (Co-expression Meta-analysis of miRNA Targets) (131) is based on the assumption that the targets of a given miRNA are likely to be co-expressed and therefore to belong to the same miRNA gene network. CoMeTa aims at inferring miRNA targets and miRNA-regulated gene networks by integrating expression data from hundreds of cell and tissue conditions. The three tools are not regularly updated and are using an older miRBase version.

### MiRNA metabolic and signaling pathway analysis

MiRNAs are functionally related both to signaling (132) and metabolic (133) networks and extensively interact with other factors (134) through distinct topological patterns, integrating transcriptional and post-transcriptional mechanisms into biological regulatory networks. Moreover, they typically have multiple targets within cellular networks that possibly enable modulation of entire pathways related to individual biological process. Despite the growing evidence for the involvement of miRNAs in central biological processes, their systematic integration in biological pathways remains incomplete. Some online tools now deal with miRNA-related pathways (Table 3). The studies that have used these tools are reported in Supporting Material S2.

**ElMMo** (135) is a Bayesian target prediction algorithm that uses evolutionary conservation and pathway analysis and can be applied to sequences from any clade of species. The algorithm automatically infers the phylogenetic distribution of functional sites for each miRNA and assigns a posterior probability to each putative target site. By combining the predictions with pathway analysis, it proposes functions of specific miRNAs in nervous system development, intercellular communication and cell growth. **MiRNApath** (136) is an online database that uses miRNA target genes to link miRNAs to metabolic pathways. Additionally, it provides five search services and a download area. There is a specific input type for each search, which may be a list of target genes, miRNAs, or metabolic pathways, and results in different outputs depending on input data. Internal links lead to a deeper level of analysis and cross-links to other databases with more detailed information. **miTALOS** (137) is a web resource providing insights into miRNA-mediated regulation of signaling pathways. It considers the tissue-specific expression signatures of miRNAs and target transcripts to improve miRNA regulation analysis in biological pathways. It identifies potential pathway regulation by (i) an enrichment analysis of miRNA target genes and (ii) using a proximity score to evaluate the functional role of miRNAs in biological pathways by their network proximity. **MiRSystem** (138) is a web-based tool providing miRNA target gene analysis, prediction of biological functions, and canonical pathways of miRNAs and their target genes. **DIANA-miRPath** (122,139,140) is a relatively efficient web-based application that performs enrichment analysis of predicted target genes of one or more miRNAs in biological pathways. It addresses the combinatorial effect of co-expressed miRNAs in the modulation of a given pathway through simultaneous analysis of multiple miRNAs. The new version of this tool performs advanced analysis pipelines, such as hierarchical clustering of miRNAs and pathways based on the levels of their interactions. Users can also easily create heat maps of miRNA-pathway interactions. The tool also provides identification of pathological single nucleotide polymorphisms (SNPs) at miRNA binding sites, as well as the ‘Reverse Search module’, where the user can identify all the predicted or experimentally validated miRNAs significantly targeting a selected pathway. Among these tools, MiRSystem and DIANA-miRPath have been updated very recently. MiRSystem and DIANA-miRPath are using miRBase v20 and v21, respectively. DIANA-miRPath includes High Confidence miRNA sets.

### MiRNA and transcription factor interaction

MiRNAs and transcription factors (TFs) are two vital classes of transregulators in gene regulatory networks. MiRNAs are important cellular components that regulate gene expression at the post-transcriptional level. It has become clear that they do not act independently, but co-operate with other molecules like TFs to regulate target genes, or execute specific functions indirectly (141). TFs are an important class of gene regulators that act at the transcriptional level. Furthermore, miRNA expression can be activated or repressed by TFs, although studies of TF-miRNA regulation are relatively limited. Recently, some databases and bioinformatic tools have been developed to gain a greater understanding of these interactions (Table 3). The studies that have used these tools are reported in Supporting Material S2.

**TransmiR** is a manually curated database that uses TF-miRNA regulatory relationships found in the literature (142). It provides a limited number of experimentally validated TF-miRNA regulations for multiple species as well as information on their involvement in tumors and miRNA-associated diseases, where available. The **PuTmiR** database focuses on the TFs that might regulate miRNAs (143). It provides a repository of putative TFs for any arbitrary human miRNA binding in the 10 kb upstream and downstream region. It also offers an interface that allows region-specific searches for a given miRNA both in upstream and downstream, to extract the list of putative TFs for human miRNAs, where the putative TFs are considered as the possible regulators of those miRNAs. **CircuitsDB** is a database devoted to identification and analysis of mixed miRNA-TF regulatory circuits in the human and mouse genomes based on bioinformatic sequence analysis (144). Specifically, the website focuses on the study of a particular type of connection between transcriptional and post-transcriptional interactions: the miRNA-TF feed-forward regulatory Loop (FFL), i.e. basic circuits where a master transcription factor regulates a miRNA and together with it a set of joint target protein-coding genes. Furthermore, this tool investigates the functional properties and disease relevance of proposed interactions with the aid of several external sources. The web application **MIR@NT@N** is based on a meta-regulation network model that illustrates interactions among transcription factors, miRNAs and protein-coding genes (145). It predicts regulatory networks and sub-networks including conserved motifs, feedback loops (FBLs) and FFLs. The main feature of this tool is that it enables to predict TF- and miRNA-mediated regulations on a genome-wide scale. MIR@NT@N facilitates the analysis of ‘omics’ data and allows detection of relevant molecular interactions and associated regulatory motifs (e.g. FFLs). The most updated database is **ChIPBase**, which integrates chromatin immunoprecipitation with next-generation DNA sequencing (ChIP-Seq) data to facilitate the comprehensive annotation and discovery of TF binding maps and transcriptional regulatory relationships of miRNAs from ChIP-Seq data (146). By analyzing millions of TF binding sites it has identified tens of thousands of TF-miRNA regulatory relationships. While databases like transmiR and CircuitsDB only assemble computationally predicted or experimentally supported TF-miRNA interactions, ChIPBase provides comprehensive TF-miRNA regulatory relationships identified from high-throughput ChIP-Seq data. Among these tools ChIPBase and TransmiR are updated relatively often. ChIPBase is currently using miRBase v17.

### Linking miRNA and disease

#### MiRNA deregulation in human diseases

It is well established that miRNA deregulation is associated with several human diseases (147–149) including various cancers (150). One way to study diseases involving miRNAs is by assembling data from independent sources. Therefore, an online knowledge base is crucial to provide up to date information. Some available databases (Table 3) already gather information about miRNA involvement in various diseases. Among them, **miR2Disease** (151) is a manually curated database offering comprehensive information on miRNA deregulation in various human diseases. Each entry contains detailed miRNA-disease relationship data, including miRNA ID, disease name, a brief description of the miRNA-disease relationship, miRNA expression pattern in the disease state, the miRNA expression detection method, experimentally verified miRNA target gene(s) and literature references. It also includes a page that allows submitting novel miRNA-disease relationships. **MiRò** (152), another online knowledge base providing miRNA-phenotype associations in humans, integrates data from various online sources including miRNA databases, ontologies, diseases and targets, into a single resource equipped with an intuitive and flexible query interface and data mining facilities. It allows associating genes and diseases based on miRNA annotations and functions, thus enabling selection of the most promising associations. **PhenomiR** (153) is a database providing information about miRNAs exhibiting a differential regulation in disease and other biological processes. **OncomiRDB** (154), a manually curated database reporting experimentally verified oncogenic and tumor-suppressing miRNAs, contains 2259 entries of cancer-related miRNA regulations that are based on direct experimental evidence from about 9000 abstracts, covering more than 300 miRNAs and 829 target genes across 25 cancer tissues. It provides both graphical and text-based interfaces that facilitate both computational analysis and experimental study of miRNA regulatory networks and functions in cancer. **miRCancer** (155) is another miRNA-cancer association database that is constructed by text mining on literature. It contains 878 relationships among 236 miRNAs and 79 human cancers obtained by processing 426 000 published articles. **HMDD** (156), Human microRNA Disease Database, collects experimentally supported human miRNA-disease association data from genetics, epigenetics, circulating miRNAs and miRNA-target interactions. In addition, it presents data generated on the basis of concepts derived from the miRNA-disease association data, including disease spectrum width of miRNAs and miRNA spectrum width of human diseases. A link for data download and one for submission of novel data to the database are also provided.

Among these databases, miR2Disease and miRò have not been updated for more than 5 years and are linked to a very old miRBase version. PhenomiR is newer, but it too is associated with an older miRBase version (v12). OncomiRDB, miRCancer and HMDD are using miRBase v20, v18 and v20, respectively. Whereas OncomiRDB and miRCancer provide only data from cancers, HMDD provides information from various human diseases. Please see Supporting Material S2 for the studies that have employed these databases.

#### Extracellular circulating miRNAs

MiRNAs found in human extracellular body fluids such as serum, plasma, saliva and urine are referred to as circulating miRNAs (46). Such miRNAs are considered as potential biomarkers, because they are easily collected, they are stable under different storage and experimental conditions, and can be detected using specific, sensitive and reproducible assays (157). Numerous miRNAs have been found in human body fluids to date. Some circulating miRNAs have recently been reported to be associated with disease conditions including cancer (158) and age-related diseases (159,160), suggesting that all types of circulating miRNAs in body fluids should be included in the study of miRNAs as biomarkers of disease. Given the importance of circulating miRNAs in biomedical research data are mounting quickly. A knowledge base of extracellular circulating miRNAs is therefore a key biomedical research tool. **MiRandola** (158) is a comprehensive database that provides manually curated classification of extracellular circulating miRNAs (see Table 3 and Supporting Material S2). Its connection to another miRNA database, miRò (152), allows users to infer the potential biological functions of circulating miRNAs and their connections with phenotypes.

#### MiRNAs, environmental factors and phenotype

MiRNAs are involved in a number of biological processes and human diseases. Extensive studies have also been conducted on the association between environmental factors (EFs) and human diseases (161,162). According to recent reports miRNAs functionally interact with various EFs such as diet, stress, smoking habits, air pollution, alcohol, drugs, viruses and radiation (163), and work synchronously to influence phenotypes and diseases, including cancer (164). Computational analysis and modeling of miRNA-EF interactions thus provides crucial insights into EF mechanism and enables identification of the miRNA signature of EFs and a greater understanding of the role of their interplay in human disease. Such investigations are still extremely limited due to the lack of a large-scale miRNA-EF interaction data set. Now, the **miREnvironment** (see Table 3 and Supporting Material S2) database provides a comprehensive collection of experimentally supported interactions among miRNAs, EFs and phenotypes (165). It incorporates more than 3857 entries, 1242 miRNAs, 394 EFs, 305 phenotypes and 24 species from 557 publications. It also has a tool performing bioinformatic analysis to predict the result of cancer treatment and associations between EFs and human disease.

#### Polymorphisms in miRNA targets associated with human diseases

SNPs can affect susceptibility to disease through gene expression regulation. MiRNAs also regulate gene expression, through post-transcriptional repression, by binding to the 3′ UTR of their target mRNA. MiRNA–mRNA binding is mainly determined by pairing of the miRNA seed sequence (nucleotides 2–7) to the complementary match sites in each mRNA target (166). SNPs at the seed sites of miRNA targets may affect the complementarity of miRNA–mRNA binding positively or negatively, thus influencing phenotypes and disease susceptibility (167). In addition to SNPs within miRNA seed sites, SNPs outside miRNA binding sites (rest of the 3′ UTR) in a gene can influence miRNA function (168,169). Several reports have addressed the association of SNPs at miRNA 3′ UTR target sites with complex conditions including cardiovascular disease, neurodegenerative disorders, hippocampal sclerosis, Parkinson disease, Tourette's syndrome, asthma, periodontal disease, tumor susceptibility and various types of cancers (170). In addition, genetic variants in miRNA genes may also have important roles by influencing miRNA maturation, which may affect disease susceptibility (167).

Even though the identification of SNPs associated with diseases is gathering pace, the underlying molecular mechanisms for the majority of disease-associated SNPs in 3′ UTRs still needs to be elucidated. Here, too, a contribution to the study of the interplay between SNPs and miRNAs and of their association with disease has come from the creation of online databases and tools (see Table 3 and Supporting Material S2).

The **Patrocles** (171) database collects DNA sequence polymorphisms in the 3′ UTR of genes that perturb miRNA-mediated gene regulation in seven vertebrate species. It also provides a tool (**Patrocles finder**) that allows users to find specific polymorphisms that may perturb miRNA-mediated gene regulation of custom target sequences. The web-based application **MicroSNiPer** (172) predicts not only the impact of an SNP on putative miRNA targets in the 3′ UTR of genes, but can also be applied to any RNA/DNA sequence of interest (5′ UTRs or open reading frames, ORFs). It predicts whether an SNP within the target site will disrupt/eliminate or enhance/create a miRNA binding site. Numerous SNPs are associated with complex diseases that have been identified by genome-wide association studies (GWAS) (173). GWAS and expression quantitative trait locus (eQTL) are powerful methods to identify genetic variants that affect disease risk and gene expression**. Mirsnpscore** (174) is a computational tool that identifies the causative SNPs associated with diseases by focusing on SNPs affecting gene regulation by miRNAs. It predicts the effects of SNPs on miRNA target sites and uses linkage disequilibrium to map the miRNA-related variants to SNPs of interest in GWAS. The online database **MirSNP** (175) collects human SNPs at predicted miRNA–mRNA binding sites, which can be combined with researchers’ own GWAS or eQTL data sets to identify the putative miRNA-related SNPs associated with diseases, thus directing subsequent functional studies. The **miRdSNP** (170) database is a comprehensive data source on disease-associated SNPs that provides robust tools to explore their distance from miRNA target sites on the 3′ UTRs of human genes. It also helps explore the molecular mechanism of gene dysregulation for disease-associated SNPs at the post**-**transcriptional level. **PolymiRTS** (176,177) offers the largest number of features. It is an integrated platform designed to analyze the functional impact of genetic polymorphisms in miRNA seed regions and at miRNA target sites. It provides links between SNPs at miRNA target sites, cis-acting eQTLs and the results of GWAS of human diseases. It also integrates data from CLASH (cross-linking, ligation and sequencing of hybrids) experiments to provide complete and accurate miRNA–mRNA interactions. Among these tools MicroSNiPer and PolymiRTS are updated relatively often and are using miRBase v19 and v20, respectively.

#### Somatic mutations in miRNAs and their target sites

Whole-genome sequencing of cancers has enabled identification of somatic mutations that distinguish normal from cancer tissue genomes. In addition to germline mutations, which disrupt miRNA targeting and play important roles in cancer, somatic mutations also need to be investigated, since whole genome sequencing data are available for several cancers (178). Some databases have been set up to provide data to investigate the impact of somatic and germline mutations on miRNA function in cancer (see Table 3 and Supporting Material S2). The **SomaMir** (179) database is linked to miRBase version v17; it contains somatic mutations that can create or disrupt miRNA target sites and integrates such mutations with germline mutations at the same target sites, genome-wide and candidate gene association studies of cancer, and functional annotations that link genes containing mutations to cancer. Additionally, the database contains a collection of germline and somatic mutations, in miRNAs and their targets, that have been experimentally shown to impact miRNA function and have been associated with cancer. Another very recent tool, **miR2GO** (180), is a web server for comparative analyses of human miRNA functions. It offers two programs: miRmut2GO, which implements a knowledge-based method to assess the functional effects of genetic and somatic mutations in miRNA seed regions, and miRpair2GO, which compares the functions of two different miRNAs based on the enriched functional annotations of their target gene sets.

#### Prediction of the cell targets of host and viral miRNAs

Like all eukaryotic organisms, viruses also encode miRNAs (91) that contribute to the complex interactions between viruses and their hosts. As viruses are habitually parasitic, viral miRNAs may target important host genes to impair the host cell defense and control host cell biogenesis. For example, Human herpes virus 4 (Epstein-Barr virus) represses a number of host genes, including those encoding B cell-specific chemokines and cytokines, transcriptional regulators and components of signal transduction pathways, using virus-encoded miRNAs (91). Even though large-scale computational prediction of miRNAs has been conducted for many organisms using known genomic sequences, data for the thousands of known viral genomes are extremely limited. However, some bioinformatic tools are available and are presented below (see Table 3 and Supporting Material S2).

**ViTa** (181) is a viral database that curates the known virus miRNA genes as well as the known/putative target sites of human, mouse, rat and chicken miRNAs. It also contains the virus annotations, virus-infected tissues and tissue specificity of host miRNAs. ViTa also facilitates comparisons between virus subtypes, such as influenza viruses, human liver viruses and the conserved regions between viruses. A similar database **Vir-Mir db** (56) predicts viral miRNA candidate hairpins. It has examined 2266 available viral genome sequences for putative miRNA hairpins and identified 33 691 hairpin candidates in 1491 genomes. **Bi-Targeting** (182) is an algorithm that identifies groups of viral and host miRNAs that cooperate in post-transcriptional gene regulation, and their target genes that are involved in similar biological processes. **RepTar** (183) is a database that provides a comprehensive set of conventional (‘seed’ type) and non-conventional miRNA target predictions, including 3′-compensatory and centered sites. It offers genome-wide predictions of cellular targets of host and viral miRNAs and provides sophisticated data-mining techniques for querying the large data set of miRNA-target predictions. However, all the tools of this category require updating, since they are out of date and are using old miRBase versions.

These bioinformatic resources are valuable tools for biomedical researchers who study virus–host interactions, to identify possible viral miRNAs and their target genes in hosts.

## CONCLUSION AND PROSPECTS

Since miRNAs are involved in a variety of biological processes and their deregulation can be linked to cancer and several other diseases, they have a huge impact on biomedical research. Despite the large number of studies carried out to date, our understanding of miRNAs and their large-scale regulatory mechanisms is still limited. High-throughput technologies have significantly advanced our knowledge of miRNAs; now bioinformatic tools are making it possible to address all the aspects of miRNA research pipelines. We have reviewed various bioinformatic resources that can be harnessed in miRNA research; they cover an impressive range, from miRNA gene and target prediction to the functional implication of miRNAs.

These tools still have a few flaws, whose correction would refine existing resources and help develop new ones. The most common and vital limitation of these computational tools is generation of large amounts of false-positive data. Machine-learning-based programs and filter-based algorithms can minimize their rate. Furthermore, integrated platforms that incorporate multiple computational tools would probably produce better outputs than a single algorithm. Integrated tools act as hubs for executable programs, enabling generation of comprehensive and reliable miRNA information.

The mounting body of NGS and gene expression data being generated requires increasingly sophisticated analytical tools. Future tools need more user-friendly features for improved efficiency; although some already have them, this would be a useful general property. Resources should also be downloadable, to allow user inputs and data processing. Open source software would also be useful for advanced users, to enable customization and improvement. Systematic updating with the update of the experimental data in the corresponding databases would be another valuable feature. Furthermore, in this increasingly open scientific age, all researchers should make their raw data freely accessible, thus enabling independent bioinformatic analysis and interpretation. Authors should therefore be allowed to upload their data and findings in online repositories in simple format.

Social networking also in the scientific community is increasing contacts among researchers around the globe. A new study or tool is immediately discussed in scientific forums throughout the world and its strengths and weaknesses highlighted, encouraging and guiding progress. The interaction between users and developers could significantly contribute to the design of more efficient bioinformatic platforms. Since miRNAs are frequently dysregulated in human disease, they are considered as promising targets for therapeutic intervention. A powerful bioinformatic platform could play a crucial role in this type of research. Next-generation biomedical research will hugely benefit from bioinformatic resources, since the massive flow of miRNA data cannot be managed without them. This overview is probably most useful to miRNA scientists, who need to master bioinformatic tools to enhance their research, but it could also inspire bioinformatics experts and resource developers to design more user-friendly next-generation miRNA tools.

## Supplementary Material

SUPPLEMENTARY DATA

## References

[1] Lee R.C., Feinbaum R.L., Ambros V. (1993). The *C. elegans* heterochronic gene lin-4 encodes small RNAs with antisense complementarity to *lin-14*. Cell.

[2] Reinhart B.J., Slack F.J., Basson M., Pasquinelli A.E., Bettinger J.C., Rougvie A.E., Horvitz H.R., Ruvkun G. (2000). The 21-nucleotide let-7 RNA regulates developmental timing in *Caenorhabditis elegans*. Nature.

[3] Bartel D.P. (2004). MicroRNAs: genomics, biogenesis, mechanism, and function. Cell.

[4] Lee Y., Kim M., Han J., Yeom K.H., Lee S., Baek S.H., Kim V.N. (2004). MicroRNA genes are transcribed by RNA polymerase II. EMBO J..

[5] Rodriguez A., Griffiths-Jones S., Ashurst J.L., Bradley A. (2004). Identification of mammalian microRNA host genes and transcription units. Genome Res..

[6] Cai X., Hagedorn C.H., Cullen B.R. (2004). Human microRNAs are processed from capped, polyadenylated transcripts that can also function as mRNAs. RNA.

[7] Kim V.N. (2005). MicroRNA biogenesis: coordinated cropping and dicing. Nat. Rev. Mol. Cell Biol..

[8] Bartel B. (2005). MicroRNAs directing siRNA biogenesis. Nat. Struct. Mol. Biol..

[9] Yates L.A., Norbury C.J., Gilbert R.J.C. (2013). The long and short of microRNA. Cell.

[10] Friedman R.C., Farh K.K.-H., Burge C.B., Bartel D.P. (2009). Most mammalian mRNAs are conserved targets of microRNAs. Genome Res..

[11] Zhang B., Pan X., Wang Q., Cobb G.P., Anderson T.A. (2006). Computational identification of microRNAs and their targets. Comput. Biol. Chem..

[12] Cho W. (2010). MicroRNAs: potential biomarkers for cancer diagnosis, prognosis and targets for therapy. Int. J. Biochem. Cell Biol..

[13] Nana-Sinkam S.P., Croce C.M. (2013). Clinical applications for microRNAs in cancer. Clin. Pharmacol. Ther..

[14] Borchert G.M., Lanier W., Davidson B.L. (2006). RNA polymerase III transcribes human microRNAs. Nat. Struct. Mol. Biol..

[15] Zeng Y., Cullen B.R. (2004). Structural requirements for pre-microRNA binding and nuclear export by Exportin 5. Nucleic Acids Res..

[16] Bohnsack M.T., Czaplinski K., GÃ-Rlich D. (2004). Exportin 5 is a RanGTP-dependent dsRNA-binding protein that mediates nuclear export of pre-miRNAs. RNA.

[17] Hutvagner G., McLachlan J., Pasquinelli A.E., Bálint É., Tuschl T., Zamore P.D. (2001). A cellular function for the RNA-interference enzyme Dicer in the maturation of the let-7 small temporal RNA. Science.

[18] Winter J., Jung S., Keller S., Gregory R.I., Diederichs S. (2009). Many roads to maturity: microRNA biogenesis pathways and their regulation. Nat. Cell Biol..

[19] Ambros V. (2001). microRNAs: tiny regulators with great potential. Cell.

[20] Ambros V. (2004). The functions of animal microRNAs. Nature.

[21] Lim L.P., Lau N.C., Garrett-Engele P., Grimson A., Schelter J.M., Castle J., Bartel D.P., Linsley P.S., Johnson J.M. (2005). Microarray analysis shows that some microRNAs downregulate large numbers of target mRNAs. Nature.

[22] Pillai R.S. (2005). MicroRNA function: multiple mechanisms for a tiny RNA?. RNA.

[23] Eiring A.M., Harb J.G., Neviani P., Garton C., Oaks J.J., Spizzo R., Liu S., Schwind S., Santhanam R., Hickey C.J. (2010). miR-328 functions as an RNA decoy to modulate hnRNP E2 regulation of mRNA translation in leukemic blasts. Cell.

[24] Kim D.H., Sætrom P.l., Snøve O., Rossi J.J. (2008). MicroRNA-directed transcriptional gene silencing in mammalian cells. Proc. Natl. Acad. Sci. U.S.A..

[25] Vasudevan S., Tong Y., Steitz J.A. (2007). Switching from repression to activation: microRNAs can up-regulate translation. Science.

[26] Gong H., Liu C.M., Liu D.P., Liang C.C. (2005). The role of small RNAs in human diseases: potential troublemaker and therapeutic tools. Med. Res. Rev..

[27] Santamaria X., Taylor H. (2014). MicroRNA and gynecological reproductive diseases. Fertil. Steril..

[28] Condorelli G., Latronico M.V.G., Cavarretta E. (2014). microRNAs in cardiovascular diseases: current knowledge and the road ahead. J. Am. Coll. Cardiol..

[29] Guay C., Roggli E., Nesca V., Jacovetti C.c., Regazzi R. (2011). Diabetes mellitus, a microRNA-related disease?. Transl. Res..

[30] Garzon R., Marcucci G., Croce C.M. (2010). Targeting microRNAs in cancer: rationale, strategies and challenges. Nat. Rev. Drug Discov..

[31] Jacobsen A., Silber J., Harinath G., Huse J.T., Schultz N., Sander C. (2013). Analysis of microRNA-target interactions across diverse cancer types. Nat. Struct. Mol. Biol..

[32] Li Z., Rana T.M. (2014). Therapeutic targeting of microRNAs: current status and future challenges. Nat. Rev. Drug Discov..

[33] Ma L., Teruya-Feldstein J., Weinberg R.A. (2007). Tumour invasion and metastasis initiated by microRNA-10b in breast cancer. Nature.

[34] Tavazoie S.F., Alarcón C., Oskarsson T., Padua D., Wang Q., Bos P.D., Gerald W.L., Massagué J. (2008). Endogenous human microRNAs that suppress breast cancer metastasis. Nature.

[35] Zhang B., Pan X., Cobb G.P., Anderson T.A. (2007). microRNAs as oncogenes and tumor suppressors. Dev. Biol..

[36] Li H., Yang B.B. (2013). Friend or foe: the role of microRNA in chemotherapy resistance. Acta Pharmacol. Sin..

[37] Calin G.A., Dumitru C.D., Shimizu M., Bichi R., Zupo S., Noch E., Aldler H., Rattan S., Keating M., Rai K. (2002). Frequent deletions and down-regulation of micro-RNA genes miR15 and miR16 at 13q14 in chronic lymphocytic leukemia. Proc. Natl. Acad. Sci. U.S.A..

[38] Takamizawa J., Konishi H., Yanagisawa K., Tomida S., Osada H., Endoh H., Harano T., Yatabe Y., Nagino M., Nimura Y. (2004). Reduced expression of the let-7 microRNAs in human lung cancers in association with shortened postoperative survival. Cancer Res..

[39] Pan X., Wang Z.-X., Wang R. (2010). MicroRNA-21: a novel therapeutic target in human cancer. Cancer Biol. Ther..

[40] Echevarría-Vargas I.M., Valiyeva F., Vivas-Mejía P.E. (2014). Upregulation of miR-21 in cisplatin resistant ovarian cancer via JNK-1/c-Jun pathway. PloS One.

[41] Felli N., Fontana L., Pelosi E., Botta R., Bonci D., Facchiano F., Liuzzi F., Lulli V., Morsilli O., Santoro S. (2005). MicroRNAs 221 and 222 inhibit normal erythropoiesis and erythroleukemic cell growth via kit receptor down-modulation. Proc. Natl. Acad. Sci. U.S.A..

[42] Pineau P., Volinia S., McJunkin K., Marchio A., Battiston C., Terris B., Mazzaferro V., Lowe S.W., Croce C.M., Dejean A. (2010). miR-221 overexpression contributes to liver tumorigenesis. Proc. Natl. Acad. Sci. U.S.A..

[43] Lawrie C.H., Gal S., Dunlop H.M., Pushkaran B., Liggins A.P., Pulford K., Banham A.H., Pezzella F., Boultwood J., Wainscoat J.S. (2008). Detection of elevated levels of tumour-associated microRNAs in serum of patients with diffuse large B-cell lymphoma. Br. J. Haematol..

[44] Cheng H., Zhang L., Cogdell D.E., Zheng H., Schetter A.J., Nykter M., Harris C.C., Chen K., Hamilton S.R., Zhang W. (2011). Circulating plasma MiR-141 is a novel biomarker for metastatic colon cancer and predicts poor prognosis. PloS One.

[45] Zheng H., Liu J.-Y., Song F.-J., Chen K.-X. (2013). Advances in circulating microRNAs as diagnostic and prognostic markers for ovarian cancer. Cancer Biol. Med..

[46] Zhao Y.-N., Chen G.-S., Hong S.-J. (2014). Circulating MicroRNAs in gynecological malignancies: from detection to prediction. Exp. Hematol. Oncol..

[47] Van Rooij E., Kauppinen S. (2014). Development of microRNA therapeutics is coming of age. EMBO Mol. Med..

[48] Thum T. (2012). MicroRNA therapeutics in cardiovascular medicine. EMBO Mol. Med..

[49] Bader A.G., Brown D., Stoudemire J., Lammers P. (2011). Developing therapeutic microRNAs for cancer. Gene Ther..

[50] Vester B., Wengel J. (2004). LNA (locked nucleic acid): high-affinity targeting of complementary RNA and DNA. Biochemistry.

[51] Ebert M.S., Neilson J.R., Sharp P.A. (2007). MicroRNA sponges: competitive inhibitors of small RNAs in mammalian cells. Nat. Methods.

[52] Choi W.-Y., Giraldez A.J., Schier A.F. (2007). Target protectors reveal dampening and balancing of Nodal agonist and antagonist by miR-430. Science.

[53] Gumireddy K., Young D.D., Xiong X., Hogenesch J.B., Huang Q., Deiters A. (2008). Small-molecule inhibitors of microRNA miR-21 function. Angew. Chem. Int. Ed. Engl..

[54] Milagro F.I., Miranda J., Portillo M.P., Fernandez-Quintela A., Campión J., Martínez J.A. (2013). High-throughput sequencing of microRNAs in peripheral blood mononuclear cells: identification of potential weight loss biomarkers. PloS One.

[55] Siomi H., Siomi M.C. (2010). Posttranscriptional regulation of microRNA biogenesis in animals. Mol. Cell.

[56] Li S.-C., Shiau C.-K., Lin W.-C. (2008). Vir-Mir db: prediction of viral microRNA candidate hairpins. Nucleic Acids Res..

[57] Kozomara A., Griffiths-Jones S. (2014). miRBase: annotating high confidence microRNAs using deep sequencing data. Nucleic Acids Res..

[58] Bentwich I., Avniel A., Karov Y., Aharonov R., Gilad S., Barad O., Barzilai A., Einat P., Einav U., Meiri E. (2005). Identification of hundreds of conserved and nonconserved human microRNAs. Nat. Genet..

[59] Várallyay É., Burgyán J., Havelda Z. (2007). Detection of microRNAs by Northern blot analyses using LNA probes. Methods.

[60] Liu C.-G., Calin G.A., Meloon B., Gamliel N., Sevignani C., Ferracin M., Dumitru C.D., Shimizu M., Zupo S., Dono M. (2004). An oligonucleotide microchip for genome-wide microRNA profiling in human and mouse tissues. Proc. Natl. Acad. Sci. U.S.A..

[61] Nelson P.T., Baldwin D.A., Kloosterman W.P., Kauppinen S., Plasterk R.H., Mourelatos Z. (2006). RAKE and LNA-ISH reveal microRNA expression and localization in archival human brain. RNA.

[62] Mendes N.D., Freitas A.T., Sagot M.F. (2009). Current tools for the identification of miRNA genes and their targets. Nucleic Acids Res..

[63] Bar M., Wyman S.K., Fritz B.R., Qi J., Garg K.S., Parkin R.K., Kroh E.M., Bendoraite A., Mitchell P.S., Nelson A.M. (2008). MicroRNA discovery and profiling in human embryonic stem cells by deep sequencing of small RNA libraries. Stem Cells.

[64] Li L., Xu J., Yang D., Tan X., Wang H. (2010). Computational approaches for microRNA studies: a review. Mamm. Genome.

[65] Lim L.P., Lau N.C., Weinstein E.G., Abdelhakim A., Yekta S., Rhoades M.W., Burge C.B., Bartel D.P. (2003). The microRNAs of Caenorhabditis elegans. Genes Dev..

[66] Lai E.C., Tomancak P., Williams R.W., Rubin G.M. (2003). Computational identification of Drosophila microRNA genes. Genome Biol..

[67] Agarwal S., Vaz C., Bhattacharya A., Srinivasan A. (2010). Prediction of novel precursor miRNAs using a context-sen- sitive hidden Markov model (CSHMM). BMC Bioinform..

[68] Mitchell T.M. (1997). Machine Learning.

[69] Yousef M., Jung S., Kossenkov A.V., Showe L.C., Showe M.K. (2007). Naïve Bayes for microRNA target predictions-machine learning for microRNA targets. Bioinformatics.

[70] Ben-Hur A., Weston J. (2010). A user's guide to support vector machines. Methods Mol. Biol..

[71] Sheng Y., Engstrom P.G., Lenhard B. (2007). Mammalian microRNA prediction through a support vector machine model of sequence and structure. PloS One.

[72] Nam J.-W., Shin K.-R., Han J., Lee Y., Kim V.N., Zhang B.-T. (2005). Human microRNA prediction through a probabilistic co-learning model of sequence and structure. Nucleic Acids Res..

[73] Nam J.-W., Kim J., Kim S.-K., Zhang B.-T. (2006). ProMiR II: a web server for the probabilistic prediction of clustered, nonclustered, conserved and nonconserved microRNAs. Nucleic Acids Res..

[74] Terai G., Komori T., Asai K., Kin T. (2007). miRRim: a novel system to find conserved miRNAs with high sensitivity and specificity. RNA.

[75] Kadri S., Hinman V., Benos P.V. (2009). HHMMiR: efficient de novo prediction of microRNAs using hierarchical hidden Markov models. BMC Bioinform..

[76] Oulas A., Boutla A., Gkirtzou K., Reczko M., Kalantidis K., Poirazi P. (2009). Prediction of novel microRNA genes in cancer-associated genomic regions - a combined computational and experimental approach. Nucleic Acids Res..

[77] Oulas A., Poirazi P. (2011). MicroRNA and Cancer.

[78] Huang T.-H., Fan B., Rothschild M.F., Hu Z.-L., Li K., Zhao S.-H. (2007). MiRFinder: an improved approach and software implementation for genome-wide fast microRNA precursor scans. BMC Bioinform..

[79] Yousef M., Nebozhyn M., Shatkay H., Kanterakis S., Showe L.C., Showe M.K. (2006). Combining multi-species genomic data for microRNA identification using a Naive Bayes classifier. Bioinformatics.

[80] Gkirtzou K., Tsamardinos I., Tsakalides P., Poirazi P. (2010). MatureBayes: a probabilistic algorithm for identifying the mature miRNA within novel precursors. PloS One.

[81] Friedländer M.R., Chen W., Adamidi C., Maaskola J., Einspanier R., Knespel S., Rajewsky N. (2008). Discovering microRNAs from deep sequencing data using miRDeep. Nat. Biotechnol..

[82] Friedländer M.R., Mackowiak S.D., Li N., Chen W., Rajewsky N. (2012). miRDeep2 accurately identifies known and hundreds of novel microRNA genes in seven animal clades. Nucleic Acids Res..

[83] Hackenberg M., Sturm M., Langenberger D., Falcon-Perez J.M., Aransay A.M. (2009). miRanalyzer: a microRNA detection and analysis tool for next-generation sequencing experiments. Nucleic Acids Res..

[84] Hackenberg M., Rodríguez-Ezpeleta N., Aransay A.M. (2011). miRanalyzer: an update on the detection and analysis of microRNAs in high-throughput sequencing experiments. Nucleic Acids Res..

[85] Jha A., Shankar R. (2013). miReader: discovering novel mirnas in species without sequenced genome. PloS One.

[86] Huang Y., Zou Q., Wang S.P., Tang S.M., Zhang G.Z., Shen X.J. (2011). The discovery approaches and detection methods of microRNAs. Mol. Biol. Rep..

[87] Lewis B.P., Burge C.B., Bartel D.P. (2005). Conserved seed pairing, often flanked by adenosines, indicates that thousands of human genes are microRNA targets. Cell.

[88] Wong N., Wang X. (2014). miRDB: an online resource for microRNA target prediction and functional annotations. Nucleic Acids Res..

[89] Maragkakis M., Reczko M., Simossis V.A., Alexiou P., Papadopoulos G.L., Dalamagas T., Giannopoulos G., Goumas G., Koukis E., Kourtis K. (2009). DIANA-microT web server: elucidating microRNA functions through target prediction. Nucleic Acids Res..

[90] Krüger J., Rehmsmeier M. (2006). RNAhybrid: microRNA target prediction easy, fast and flexible. Nucleic Acids Res..

[91] Pfeffer S.b., Zavolan M., Grässer F.A., Chien M., Russo J.J., Ju J., John B., Enright A.J., Marks D., Sander C. (2004). Identification of virus-encoded microRNAs. Science.

[92] Thomson D.W., Bracken C.P., Goodall G.J. (2011). Experimental strategies for microRNA target identification. Nucleic Acids Res..

[93] Zhang Y. (2005). miRU: an automated plant miRNA target prediction server. Nucleic Acids Res..

[94] Bartel D.P. (2009). MicroRNAs: target recognition and regulatory functions. Cell.

[95] Shirdel E.A., Xie W., Mak T.W., Jurisica I. (2011). NAViGaTing the micronome - using multiple microRNA prediction databases to identify signalling pathway-associated microRNAs. PloS One.

[96] Rajewsky N. (2006). microRNA target predictions in animals. Nat. Genet..

[97] Maziere P., Enright A.J. (2007). Prediction of microRNA targets. Drug Discov. Today.

[98] Agarwal V., Bell G.W., Nam J.-W., Bartel D.P. (2015). Predicting effective microRNA target sites in mammalian mRNAs. Elife.

[99] Lall S., Grün D., Krek A., Chen K., Wang Y.-L., Dewey C.N., Sood P., Colombo T., Bray N., MacMenamin P. (2006). A genome-wide map of conserved microRNA targets in C. elegans. Curr. Biol..

[100] Rehmsmeier M., Steffen P., Höchsmann M., Giegerich R. (2004). Fast and effective prediction of microRNA/target duplexes. RNA.

[101] Miranda K.C., Huynh T., Tay Y., Ang Y.-S., Tam W.-L., Thomson A.M., Lim B., Rigoutsos I. (2006). A pattern-based method for the identification of MicroRNA binding sites and their corresponding heteroduplexes. Cell.

[102] Loher P., Rigoutsos I. (2012). Interactive exploration of RNA22 microRNA target predictions. Bioinformatics.

[103] Kertesz M., Iovino N., Unnerstall U., Gaul U., Segal E. (2007). The role of site accessibility in microRNA target recognition. Nat. Genet..

[104] Wang X. (2008). miRDB: a microRNA target prediction and functional annotation database with a wiki interface. RNA.

[105] Betel D., Koppal A., Agius P., Sander C., Leslie C. (2010). Comprehensive modeling of microRNA targets predicts functional non-conserved and non-canonical sites. Genome Biol..

[106] Maragkakis M., Vergoulis T., Alexiou P., Reczko M., Plomaritou K., Gousis M., Kourtis K., Koziris N., Dalamagas T., Hatzigeorgiou A.G. (2011). DIANA-microT web server upgrade supports Fly and Worm miRNA target prediction and bibliographic miRNA to disease association. Nucleic Acids Res..

[107] Paraskevopoulou M.D., Georgakilas G., Kostoulas N., Vlachos I.S., Vergoulis T., Reczko M., Filippidis C., Dalamagas T., Hatzigeorgiou A.G. (2013). DIANA-microT web server v5. 0: service integration into miRNA functional analysis workflows. Nucleic Acids Res..

[108] Hafner M., Landthaler M., Burger L., Khorshid M., Hausser J., Berninger P., Rothballer A., Ascano M., Jungkamp A.-C., Munschauer M. (2010). Transcriptome-wide identification of RNA-binding protein and microRNA target sites by PAR-CLIP. Cell.

[109] Rennie W., Liu C., Carmack C.S., Wolenc A., Kanoria S., Lu J., Long D., Ding Y. (2014). STarMir: a web server for prediction of microRNA binding sites. Nucleic Acids Res..

[110] Liu C., Mallick B., Long D., Rennie W.A., Wolenc A., Carmack C.S., Ding Y. (2013). CLIP-based prediction of mammalian microRNA binding sites. Nucleic Acids Res..

[111] Farh K.K.-H., Grimson A., Jan C., Lewis B.P., Johnston W.K., Lim L.P., Burge C.B., Bartel D.P. (2005). The widespread impact of mammalian MicroRNAs on mRNA repression and evolution. Science.

[112] Reczko M., Maragkakis M., Alexiou P., Grosse I., Hatzigeorgiou A.G. (2012). Functional microRNA targets in protein coding sequences. Bioinformatics.

[113] Vergoulis T., Vlachos I.S., Alexiou P., Georgakilas G., Maragkakis M., Reczko M., Gerangelos S., Koziris N., Dalamagas T., Hatzigeorgiou A.G. (2012). TarBase 6.0: capturing the exponential growth of miRNA targets with experimental support. Nucleic Acids Res..

[114] Vlachos I.S., Paraskevopoulou M.D., Karagkouni D., Georgakilas G., Vergoulis T., Kanellos I., Anastasopoulos I.-L., Maniou S., Karathanou K., Kalfakakou D. (2015). DIANA-TarBase v7. 0: indexing more than half a million experimentally supported miRNA: mRNA interactions. Nucleic Acids Res..

[115] Hsu S.-D., Lin F.-M., Wu W.-Y., Liang C., Huang W.-C., Chan W.-L., Tsai W.-T., Chen G.-Z., Lee C.-J., Chiu C.-M. (2010). miRTarBase: a database curates experimentally validated microRNA-target interactions. Nucleic Acids Res..

[116] Hsu S.-D., Tseng Y.-T., Shrestha S., Lin Y.-L., Khaleel A., Chou C.-H., Chu C.-F., Huang H.-Y., Lin C.-M., Ho S.-Y. (2014). miRTarBase update 2014: an information resource for experimentally validated miRNA-target interactions. Nucleic Acids Res..

[117] Xiao F., Zuo Z., Cai G., Kang S., Gao X., Li T. (2009). miRecords: an integrated resource for microRNA-target interactions. Nucleic Acids Res..

[118] Yang J.-H., Li J.-H., Shao P., Zhou H., Chen Y.-Q., Qu L.-H. (2011). starBase: a database for exploring microRNA-mRNA interaction maps from Argonaute CLIP-Seq and Degradome-Seq data. Nucleic Acids Res..

[119] Li J.-H., Liu S., Zhou H., Qu L.-H., Yang J.-H. (2014). starBase v2. 0: decoding miRNA-ceRNA, miRNA-ncRNA and protein-RNA interaction networks from large-scale CLIP-Seq data. Nucleic Acids Res..

[120] Le Brigand K., Robbe-Sermesant K., Mari B., Barbry P. (2010). MiRonTop: mining microRNAs targets across large scale gene expression studies. Bioinformatics.

[121] Alexiou P., Maragkakis M., Papadopoulos G.L., Simmosis V.A., Zhang L., Hatzigeorgiou A.G. (2010). The DIANA-mirExTra web server: from gene expression data to microRNA function. PloS One.

[122] Vlachos I.S., Kostoulas N., Vergoulis T., Georgakilas G., Reczko M., Maragkakis M., Paraskevopoulou M.D., Prionidis K., Dalamagas T., Hatzigeorgiou A.G. (2012). DIANA miRPath v. 2.0: investigating the combinatorial effect of microRNAs in pathways. Nucleic Acids Res..

[123] Kaya K.D., Karakülah G.k., Yakıcıer C.M., Acar A.C., Konu O.z. (2011). mESAdb: microRNA expression and sequence analysis database. Nucleic Acids Res..

[124] Khoury M.J., Dorman J.S. (1998). The human genome epidemiology network. Am. J. Epidemiol..

[125] Kanehisa M., Goto S. (2000). KEGG: kyoto encyclopedia of genes and genomes. Nucleic Acids Res..

[126] Ashburner M., Ball C.A., Blake J.A., Botstein D., Butler H., Cherry J.M., Davis A.P., Dolinski K., Dwight S.S., Eppig J.T. (2000). Gene Ontology: tool for the unification of biology. Nat. Genet..

[127] Cho S., Jang I., Jun Y., Yoon S., Ko M., Kwon Y., Choi I., Chang H., Ryu D., Lee B. (2013). MiRGator v3. 0: a microRNA portal for deep sequencing, expression profiling and mRNA targeting. Nucleic Acids Res..

[128] Sales G., Coppe A., Bisognin A., Biasiolo M., Bortoluzzi S., Romualdi C. (2010). MAGIA, a web-based tool for miRNA and Genes Integrated Analysis. Nucleic Acids Res..

[129] Huang D.W., Sherman B.T., Lempicki R.A. (2008). Systematic and integrative analysis of large gene lists using DAVID bioinformatics resources. Nat. Protoc..

[130] Huang G.T., Athanassiou C., Benos P.V. (2011). mirConnX: condition-specific mRNA-microRNA network integrator. Nucleic Acids Res..

[131] Gennarino V.A., D'Angelo G., Dharmalingam G., Fernandez S., Russolillo G., Sanges R., Mutarelli M., Belcastro V., Ballabio A., Verde P. (2012). Identification of microRNA-regulated gene networks by expression analysis of target genes. Genome Res..

[132] Cui Q., Yu Z., Purisima E.O., Wang E. (2006). Principles of microRNA regulation of a human cellular signaling network. Mol. Syst. Biol..

[133] Tibiche C., Wang E. (2008). MicroRNA regulatory patterns on the human metabolic network. Open Syst. Biol. J..

[134] Yu X., Lin J., Zack D.J., Mendell J.T., Qian J. (2008). Analysis of regulatory network topology reveals functionally distinct classes of microRNAs. Nucleic Acids Res..

[135] Gaidatzis D., van Nimwegen E., Hausser J., Zavolan M. (2007). Inference of miRNA targets using evolutionary conservation and pathway analysis. BMC Bioinform..

[136] Chiromatzo A.O., Oliveira T.Y., Pereira G., Costa A.Y., Montesco C.A., Gras D.E., Yosetake F., Vilar J.B., Cervato M., Prado P.R. (2007). miRNApath: a database of miRNAs, target genes and metabolic pathways. Genet. Mol. Res..

[137] Kowarsch A., Preusse M., Marr C., Theis F.J. (2011). miTALOS: analyzing the tissue-specific regulation of signaling pathways by human and mouse microRNAs. RNA.

[138] Lu T.-P., Lee C.-Y., Tsai M.-H., Chiu Y.-C., Hsiao C.K., Lai L.-C., Chuang E.Y. (2012). miRSystem: an integrated system for characterizing enriched functions and pathways of microRNA targets. PloS One.

[139] Papadopoulos G.L., Alexiou P., Maragkakis M., Reczko M., Hatzigeorgiou A.G. (2009). DIANA-mirPath: Integrating human and mouse microRNAs in pathways. Bioinformatics.

[140] Vlachos I.S., Zagganas K., Paraskevopoulou M.D., Georgakilas G., Karagkouni D., Vergoulis T., Dalamagas T., Hatzigeorgiou A.G. (2015). DIANA-miRPath v3. 0: deciphering microRNA function with experimental support. Nucleic Acids Res..

[141] Martinez N.J., Walhout A.J.M. (2009). The interplay between transcription factors and microRNAs in genome-scale regulatory networks. Bioessays.

[142] Wang J., Lu M., Qiu C., Cui Q. (2010). TransmiR: a transcription factor-microRNA regulation database. Nucleic Acids Res..

[143] Bandyopadhyay S., Bhattacharyya M. (2010). PuTmiR: a database for extracting neighboring transcription factors of human microRNAs. BMC Bioinform..

[144] Friard O., Re A., Taverna D., De Bortoli M., Corá D. (2010). CircuitsDB: a database of mixed microRNA/transcription factor feed-forward regulatory circuits in human and mouse. BMC Bioinform..

[145] Le Béchec A., Portales-Casamar E., Vetter G., Moes M., Zindy P.-J., Saumet A., Arenillas D., Theillet C., Wasserman W.W., Lecellier C.-H. (2011). MIR@ NT@ N: a framework integrating transcription factors, microRNAs and their targets to identify sub-network motifs in a meta-regulation network model. BMC Bioinform..

[146] Yang J.-H., Li J.-H., Jiang S., Zhou H., Qu L.-H. (2013). ChIPBase: a database for decoding the transcriptional regulation of long non-coding RNA and microRNA genes from ChIP-Seq data. Nucleic Acids Res..

[147] Thum T., Gross C., Fiedler J., Fischer T., Kissler S., Bussen M., Galuppo P., Just S., Rottbauer W., Frantz S. (2008). MicroRNA-21 contributes to myocardial disease by stimulating MAP kinase signalling in fibroblasts. Nature.

[148] Lorenzen J.M., Haller H., Thum T. (2011). MicroRNAs as mediators and therapeutic targets in chronic kidney disease. Nat. Rev. Nephrol..

[149] Rebane A., Akdis C.A. (2014). MicroRNAs in allergy and asthma. Curr. Allergy Asthma Rep..

[150] Calin G.A., Croce C.M. (2006). MicroRNA signatures in human cancers. Nat. Rev. Cancer.

[151] Jiang Q., Wang Y., Hao Y., Juan L., Teng M., Zhang X., Li M., Wang G., Liu Y. (2009). miR2Disease: a manually curated database for microRNA deregulation in human disease. Nucleic Acids Res..

[152] Laganà A., Forte S., Giudice A., Arena M.R., Puglisi P.L., Giugno R., Pulvirenti A., Shasha D., Ferro A. (2009). miRò: a miRNA knowledge base. Database.

[153] Ruepp A., Kowarsch A., Schmidl D., Buggenthin F., Brauner B., Dunger I., Fobo G., Frishman G., Montrone C., Theis F.J. (2010). PhenomiR: a knowledgebase for microRNA expression in diseases and biological processes. Genome Biol..

[154] Wang D., Gu J., Wang T., Ding Z. (2014). OncomiRDB: a database for the experimentally verified oncogenic and tumor-suppressive microRNAs. Bioinformatics.

[155] Xie B., Ding Q., Han H., Wu D. (2013). miRCancer: a microRNA-cancer association database constructed by text mining on literature. Bioinformatics.

[156] Li Y., Qiu C., Tu J., Geng B., Yang J., Jiang T., Cui Q. (2014). HMDD v2. 0: a database for experimentally supported human microRNA and disease associations. Nucleic Acids Res..

[157] Guay C., Regazzi R. (2013). Circulating microRNAs as novel biomarkers for diabetes mellitus. Nat. Rev. Endocrinol..

[158] Russo F., Di Bella S., Nigita G., Macca V., Lagana A., Giugno R., Pulvirenti A., Ferro A. (2012). miRandola: extracellular circulating microRNAs database. PLoS One.

[159] Olivieri F., Rippo M.R., Monsurrò V., Salvioli S., Capri M., Procopio A.D., Franceschi C. (2013). MicroRNAs linking inflamm-aging, cellular senescence and cancer. Ageing Res. Rev..

[160] Olivieri F., Antonicelli R., Spazzafumo L., Santini G., Rippo M.R., Galeazzi R., Giovagnetti S., D'Alessandra Y., Marcheselli F., Capogrossi M.C. (2014). Admission levels of circulating miR-499–5p and risk of death in elderly patients after acute non-ST elevation myocardial infarction. Int. J. Cardiol..

[161] Mills N.L., Donaldson K., Hadoke P.W., Boon N.A., MacNee W., Cassee F.R., Sandström T., Blomberg A., Newby D.E. (2008). Adverse cardiovascular effects of air pollution. Nat. Clin. Pract. Cardiovasc. Med..

[162] Soto A.M., Sonnenschein C. (2010). Environmental causes of cancer: endocrine disruptors as carcinogens. Nat. Rev. Endocrinol..

[163] Chen X., Liu M.-X., Cui Q.-H., Yan G.-Y. (2012). Prediction of disease-related interactions between microRNAs and environmental factors based on a semi-supervised classifier. PloS One.

[164] Izzotti A., Pulliero A. (2014). The effects of environmental chemical carcinogens on the microRNA machinery. Int. J. Hyg. Environ. Health.

[165] Yang Q., Qiu C., Yang J., Wu Q., Cui Q. (2011). miREnvironment database: providing a bridge for microRNAs, environmental factors and phenotypes. Bioinformatics.

[166] Brennecke J., Stark A., Russell R.B., Cohen S.M. (2005). Principles of microRNA-target recognition. PLoS Biol..

[167] Ryan B., Robles A.I., Harris C.C. (2010). Genetic variation in microRNA networks: the implications for cancer research. Nat. Rev. Cancer.

[168] Mishra P.J., Humeniuk R., Mishra P.J., Longo-Sorbello G.S.A., Banerjee D., Bertino J.R. (2007). A miR-24 microRNA binding-site polymorphism in dihydrofolate reductase gene leads to methotrexate resistance. Proc. Natl. Acad. Sci. U.S.A..

[169] Hu Z., Bruno A.E. (2011). The Influence of 3′UTRs on MicroRNA function inferred from human SNP data. Comp. Funct. Genomics.

[170] Bruno A.E., Li L., Kalabus J.L., Pan Y., Yu A., Hu Z. (2012). miRdSNP: a database of disease-associated SNPs and microRNA target sites on 3′ UTRs of human genes. BMC Genomics.

[171] Hiard S., Charlier C., Coppieters W., Georges M., Baurain D. (2010). Patrocles: a database of polymorphic miRNA-mediated gene regulation in vertebrates. Nucleic Acids Res..

[172] Barenboim M., Zoltick B.J., Guo Y., Weinberger D.R. (2010). MicroSNiPer: a web tool for prediction of SNP effects on putative microRNA targets. Hum. Mutat..

[173] Hirschhorn J.N., Daly M.J. (2005). Genome-wide association studies for common diseases and complex traits. Nat. Rev. Genet..

[174] Thomas L.F., Saito T., Sætrom P.l. (2011). Inferring causative variants in microRNA target sites. Nucleic Acids Res..

[175] Liu C., Zhang F., Li T., Lu M., Wang L., Yue W., Zhang D. (2012). MirSNP, a database of polymorphisms altering miRNA target sites, identifies miRNA-related SNPs in GWAS SNPs and eQTLs. BMC Genomics.

[176] Ziebarth J.D., Bhattacharya A., Chen A., Cui Y. (2012). PolymiRTS Database 2.0: linking polymorphisms in microRNA target sites with human diseases and complex traits. Nucleic Acids Res..

[177] Bhattacharya A., Ziebarth J.D., Cui Y. (2014). PolymiRTS Database 3.0: linking polymorphisms in microRNAs and their target sites with human diseases and biological pathways. Nucleic Acids Res..

[178] Ziebarth J.D., Bhattacharya A., Cui Y. (2012). Integrative analysis of somatic mutations altering microRNA targeting in cancer genomes. PLoS One.

[179] Bhattacharya A., Ziebarth J.D., Cui Y. (2012). SomamiR: a database for somatic mutations impacting microRNA function in cancer. Nucleic Acids Res..

[180] Bhattacharya A., Cui Y. (2015). miR2GO: comparative functional analysis for microRNAs. Bioinformatics.

[181] Hsu P.W.-C., Lin L.-Z., Hsu S.-D., Hsu J.B.-K., Huang H.-D. (2007). ViTa: prediction of host microRNAs targets on viruses. Nucleic Acids Res..

[182] Veksler-Lublinsky I., Shemer-Avni Y., Kedem K., Ziv-Ukelson M. (2010). Gene bi-targeting by viral and human miRNAs. BMC Bioinform..

[183] Elefant N., Berger A., Shein H., Hofree M., Margalit H., Altuvia Y. (2011). RepTar: a database of predicted cellular targets of host and viral miRNAs. Nucleic Acids Res..

[184] Betel D., Wilson M., Gabow A., Marks D.S., Sander C. (2008). The microRNA. org resource: targets and expression. Nucleic Acids Res..

[185] Hsu P.W.C., Huang H.-D., Hsu S.-D., Lin L.-Z., Tsou A.-P., Tseng C.-P., Stadler P.F., Washietl S., Hofacker I.L. (2006). miRNAMap: genomic maps of microRNA genes and their target genes in mammalian genomes. Nucleic Acids Res..

[186] Hsu S.-D., Chu C.-H., Tsou A.-P., Chen S.-J., Chen H.-C., Hsu P.W.-C., Wong Y.-H., Chen Y.-H., Chen G.-H., Huang H.-D. (2008). miRNAMap 2.0: genomic maps of microRNAs in metazoan genomes. Nucleic Acids Res..

[187] Friedman Y., Naamati G., Linial M. (2010). MiRror: a combinatorial analysis web tool for ensembles of microRNAs and their targets. Bioinformatics.

[188] Friedman Y., Karsenty S., Linial M. (2014). miRror-Suite: decoding coordinated regulation by microRNAs. Database.

[189] Hsu J.B.K., Chiu C.-M., Hsu S.-D., Huang W.-Y., Chien C.-H., Lee T.-Y., Huang H.-D. (2011). miRTar: an integrated system for identifying miRNA-target interactions in human. BMC Bioinform..

[190] Dweep H., Sticht C., Pandey P., Gretz N. (2011). miRWalk - database: prediction of possible miRNA binding sites by ‘walking’ the genes of three genomes. J. Biomed. Inform..

[191] Coronnello C., Benos P.V. (2013). ComiR: combinatorial microRNA target prediction tool. Nucleic Acids Res..

[192] Wang P., Ning S., Wang Q., Li R., Ye J., Zhao Z., Li Y., Huang T., Li X. (2013). mirTarPri: improved prioritization of microrna targets through incorporation of functional genomics data. PloS One.

[193] Vejnar C.E., Zdobnov E.M. (2012). miRmap: comprehensive prediction of microRNA target repression strength. Nucleic Acids Res..

[194] Vejnar C.E., Blum M., Zdobnov E.M. (2013). miRmap web: comprehensive microRNA target prediction online. Nucleic Acids Res..

[195] Wu C., Bardes E.E., Jegga A.G., Aronow B.J. (2014). ToppMiR: ranking microRNAs and their mRNA targets based on biological functions and context. Nucleic Acids Res..

[196] Ahmadi H., Ahmadi A., Azimzadeh-Jamalkandi S., Shoorehdeli M.A., Salehzadeh-Yazdi A., Bidkhori G., Masoudi-Nejad A. (2013). HomoTarget: a new algorithm for prediction of microRNA targets in *Homo sapiens*. Genomics.

[197] Zhang Y., Verbeek F.J. (2010). Comparison and integration of target prediction algorithms for microRNA studies. J. Integr. Bioinform..

