# Supplementary material for: Bioinformatic tools for microRNA dissection
Source: Nucleic Acids Res. 2015 Nov 17;44(1):24–44. doi: 10.1093/nar/gkv1221 (PMC4705652; doi:10.1093/nar/gkv1221)
Supplement: SUPPLEMENTARY DATA [file supp_44_1_24__index.html]

Bioinformatic tools for microRNA dissection — SUPPLEMENTARY DATA 

# Bioinformatic tools for microRNA dissection

## SUPPLEMENTARY DATA

- SUPPLEMENTARY DATA
- SUPPLEMENTARY DATA
- SUPPLEMENTARY DATA
- SUPPLEMENTARY DATA
