# Supplementary material for: Bioinformatic tools for microRNA dissection
Source: Nucleic Acids Res. 2015 Nov 17;44(1):24–44. doi: 10.1093/nar/gkv1221 (PMC4705652; doi:10.1093/nar/gkv1221)
Supplement: SUPPLEMENTARY DATA [file supp_gkv1221_nar-03711-survey-d-2014-File005.pdf]

Supporting material S1:

List of miRNAs discovered *via* traditional evolutionary conservation approaches and machine learning-based techniques

Category	Tools	No. of potential predictions	No. of known miRNAs predicted (no. of reference/ trainng miRNAs)	No. of novel miRNA gene/ candidate	No. of experimentally verified miRNA	Organism	Reference
Comparative methods	MiRscan	36000[h]	58 (58)	30	88	Worm	(1)
	miRseeker	124	18 (24)	48[c]	24	Fly	(2)
Machine learning	ProMir	817[p]	24 (68)	23[c]		Human	(3)
	MiRRim	545[h]	212 (290)	1	1*	Human	(4)
	HHMMiR	-	85%[a]	-	-	7 species	(5)
	SSCprofiler	-	94 (98)	5862 [c]	4	Human	(6)
	MiRFinder	222[p]	60 (86)	-	-	Chicken/ human pair-wise genome alignments	(7)
	BayesMiRNA Find	218	135 (135)	83	-	Mouse	(8)
	MatureBayes	-	27:89%[b]	-	-	Human/mouse	(9)

[h]hairpin; [p]Pre-miRNA; [c]Candidate miRNA gene
* Registered in miRBase
[a] Predicted 85% of known miRNA precursors in seven species
[b] percentage of predicted candidate that match the actual miRNA start positions

References

1. Lim, L.P., Lau, N.C., Weinstein, E.G., Abdelhakim, A., Yekta, S., Rhoades, M.W., Burge, C.B. and Bartel, D.P. (2003) The microRNAs of Caenorhabditis elegans. *Genes & development*, **17**, 991-1008.
2. Lai, E.C., Tomancak, P., Williams, R.W. and Rubin, G.M. (2003) Computational identification of Drosophila microRNA genes. *Genome Biol*, **4**, R42.
3. Nam, J.-W., Shin, K.-R., Han, J., Lee, Y., Kim, V.N. and Zhang, B.-T. (2005) Human microRNA prediction through a probabilistic co-learning model of sequence and structure. *Nucleic acids research*, **33**, 3570-3581.
4. Terai, G., Komori, T., Asai, K. and Kin, T. (2007) miRRim: a novel system to find conserved miRNAs with high sensitivity and specificity. *Rna*, **13**, 2081-2090.
5. Osanto, S., Qin, Y., Buermans, H.P., Berkers, J., Lerut, E., Goeman, J.J. and Van Poppel, H. (2012) Genome-wide microRNA expression analysis of clear cell renal cell carcinoma by next generation deep sequencing. *PloS one*, **7**, e38298.
6. Oulas, A., Boutla, A., Gkirtzou, K., Reczko, M., Kalantidis, K. and Poirazi, P. (2009) Prediction of novel microRNA genes in cancer-associated genomic regions-a combined computational and experimental approach. *Nucleic acids research*, **37**, 3276-3287.
7. Huang, T.-H., Fan, B., Rothschild, M.F., Hu, Z.-L., Li, K. and Zhao, S.-H. (2007) MiRFinder: an improved approach and software implementation for genome-wide fast microRNA precursor scans. *BMC bioinformatics*, **8**, 341.
8. Yousef, M., Nebozhyn, M., Shatkay, H., Kanterakis, S., Showe, L.C. and Showe, M.K. (2006) Combining multi-species genomic data for microRNA identification using a Naive Bayes classifier. *Bioinformatics*, **22**, 1325-1334.
9. Gkirtzou, K., Tsamardinos, I., Tsakalides, P. and Poirazi, P. (2010) MatureBayes: a probabilistic algorithm for identifying the mature miRNA within novel precursors. *PloS one*, **5**, e11843.
