# Supplementary material for: Bioinformatic tools for microRNA dissection
Source: Nucleic Acids Res. 2015 Nov 17;44(1):24–44. doi: 10.1093/nar/gkv1221 (PMC4705652; doi:10.1093/nar/gkv1221)
Supplement: SUPPLEMENTARY DATA [file supp_gkv1221_nar-03711-survey-d-2014-File006.pdf]

Supporting material S2:

Table reports the application of some selected tools for microRNA discovery

Category	Tool	Application
Comparative methods	MiRscan	(1)
	miRseeker	(2)
Machine learning	ProMir	(3,4)
	MiRRim	(5)
	HHMMiR	(6)
	SSCprofiler	-
	MiRFinder	(7)
	BosFinder	-
	BayesMiRNA Find	(8)
	MatureBayes	(9-11)
NGS based	miRDeep	(12-14)
	miRanalyzer	(15,16)
	miReader	-

Table reports the application of some selected tools for microRNA target prediction

Category	Tools	Application
Single platform	TargetScan	(17,18)
	RNAhybrid	(19,20)
	PicTar	(18,21,22)
	rna22	(22,23)
	PITA	(24,25)
	miRDB	(22,26,27)
	microRNA.org	(27-29)
	DIANA-microT-CDS	(30,31)
	STarMir	(32,33)
Integrated platform	miRNAMap	(34)
	MiRror	(35-38)
	miRTar	(39)
	miRWalk	(22,40,41)
	mirDIP	(42,43)
	ComiR	(44)
	mirTarPri	(45)
	miRmap	(46)
	ToppMiR	-

Table reports the application of some bioinformatic resources to deal with different aspects of microRNA related research

**Category**	**Tools**	**Application**
Finding validated miRNA information	DIANA-TarBase	(47)
	miRTarBase	(22,48)
	miRecords	(48,49)
	StarBase	(50)
Correlating miRNA and mRNA expression	MiRonTop	(51,52)
	DIANA-mirExTra	(53)
	mESAdb	(54)
	miRGator	(55,56)
miRNA regulatory network identification	MAGIA	(57)
	mirConnX	(58)
	CoMeTa	(59)
miRNA metabolic and signaling pathway analysis	ElMMo	(60)
	miRNApath	(61)
	miTALOS	(62,63)
	miRSystem	(64,65)
	DIANA-miRPath	(66,67)
miRNA and transcription factor interaction	TransmiR	(68,69)
	PuTmiR	(70)
	CircuitsDB	(71)
	MIR@NT@N	(72,73)
	ChIPBase	(69,74)
miRNA deregulation in human disease	miR2Disease	(75)
	miRò	(76)
	PhenomiR	(77,78)
	OncomiRDB	(79)
	miRCancer	(79)
	HMDD	(77,78)
Extracellular circulating miRNA	miRandola	(80)
Linking miRNA, environmental factors and phenotype	miREnvironment	(81)
Linking polymorphisms in miRNA target with human disease	Patrocles	(82,83)
	MicroSNiPer	(84,85)
	Mirsnpscore	(80,86,87)
	MirSNP	(88,89)
	miRdSNP	(90,91)
	PolymiRTS	(89,92)
Somatic mutations in miRNAs and their target sites	SomamiR	(93)
	miR2GO	-

Prediction of cellular target of host and viral miRNA	ViTa	(94)
	Vir-Mir db	(95)
	Bi-Targeting	(96)
	RepTar	(97)

References

1. Lewis, B.P., Shih, I.h., Jones-Rhoades, M.W., Bartel, D.P. and Burge, C.B. (2003) Prediction of mammalian microRNA targets. *Cell*, **115**, 787-798.
2. Doran, J. and Strauss, W.M. (2007) Bio-informatic trends for the determination of miRNA-target interactions in mammals. *DNA and cell biology*, **26**, 353-360.
3. Smith, J.J., Putta, S., Zhu, W., Pao, G.M., Verma, I.M., Hunter, T., Bryant, S.V., Gardiner, D.M., Harkins, T.T. and Voss, S.R. (2009) Genic regions of a large salamander genome contain long introns and novel genes. *BMC Genomics*, **10**, 19.
4. Fanous, A.H., Zhao, Z., van den Oord, E.J., Maher, B.S., Thiselton, D.L., Bergen, S.E., Wormley, B., Bigdeli, T., Amdur, R.L., O'Neill, F.A. *et al.* (2010) Association study of SNAP25 and schizophrenia in Irish family and case-control samples. *Am J Med Genet B Neuropsychiatr Genet*, **153B**, 663-674.
5. Guastadisegni, M.C., Lonoce, A., Impera, L., Albano, F., D'Addabbo, P., Caruso, S., Vasta, I., Panagopoulos, I., Leszl, A. and Basso, G. (2008) Bone marrow ectopic expression of a non-coding RNA in childhood T-cell acute lymphoblastic leukemia with a novel t (2; 11)(q11. 2; p15. 1) translocation. *Mol Cancer*, **7**, 80.
6. Osanto, S., Qin, Y., Buermans, H.P., Berkers, J., Lerut, E., Goeman, J.J. and van Poppel, H. (2012) Genome-wide microRNA expression analysis of clear cell renal cell carcinoma by next generation deep sequencing. *PLoS One*, **7**, e38298.
7. Saito, T. and Rehmsmeier, M. (2015) The precision-recall plot is more informative than the ROC plot when evaluating binary classifiers on imbalanced datasets. *PLoS One*, **10**, e0118432.
8. Kumar, S., Ansari, F.A. and Scaria, V. (2009) Prediction of viral microRNA precursors based on human microRNA precursor sequence and structural features. *Virology journal*, **6**, 129.
9. Shi, J., Duan, Z., Sun, J., Wu, M., Wang, B., Zhang, J., Wang, H., Hu, N. and Hu, Y. (2014) Identification and validation of a novel microRNA-like molecule derived from a cytoplasmic RNA virus antigenome by bioinformatics and experimental approaches. *Virol J*, **11**, 121.
10. Parsi, S., Soltani, B.M., Hosseini, E., Tousi, S.E. and Mowla, S.J. (2012) Experimental verification of a predicted intronic microRNA in human NGFR gene with a potential pro-apoptotic function. *PLoS One*, **7**, e35561.
11. Severino, P., Oliveira, L.S., Torres, N., Andreghetto, F.M., Klingbeil Mde, F., Moyses, R., Wunsch-Filho, V., Nunes, F.D., Mathor, M.B., Paschoal, A.R. *et al.* (2013) High-throughput sequencing of small RNA transcriptomes reveals critical biological features targeted by microRNAs in cell models used for squamous cell cancer research. *BMC Genomics*, **14**, 735.

12. Swaminathan, S., Hu, X., Zheng, X., Kriga, Y., Shetty, J., Zhao, Y., Stephens, R., Tran, B., Baseler, M.W., Yang, J. *et al.* (2013) Interleukin-27 treated human macrophages induce the expression of novel microRNAs which may mediate anti-viral properties. *Biochem Biophys Res Commun*, **434**, 228-234.
13. Liao, J.Y., Ma, L.M., Guo, Y.H., Zhang, Y.C., Zhou, H., Shao, P., Chen, Y.Q. and Qu, L.H. (2010) Deep sequencing of human nuclear and cytoplasmic small RNAs reveals an unexpectedly complex subcellular distribution of miRNAs and tRNA 3' trailers. *PLoS One*, **5**, e10563.
14. Sharbati, S., Friedlander, M.R., Sharbati, J., Hoeke, L., Chen, W., Keller, A., Stahler, P.F., Rajewsky, N. and Einspanier, R. (2010) Deciphering the porcine intestinal microRNA transcriptome. *BMC Genomics*, **11**, 275.
15. Meng, F., Hackenberg, M., Li, Z., Yan, J. and Chen, T. (2012) Discovery of novel microRNAs in rat kidney using next generation sequencing and microarray validation. *PLoS One*, **7**, e34394.
16. Tandon, M., Gallo, A., Jang, S.I., Illei, G.G. and Alevizos, I. (2012) Deep sequencing of short RNAs reveals novel microRNAs in minor salivary glands of patients with Sjogren's syndrome. *Oral Dis*, **18**, 127-131.
17. Farh, K.K., Grimson, A., Jan, C., Lewis, B.P., Johnston, W.K., Lim, L.P., Burge, C.B. and Bartel, D.P. (2005) The widespread impact of mammalian MicroRNAs on mRNA repression and evolution. *Science*, **310**, 1817-1821.
18. Yang, Y., Sun, W., Wang, R., Lei, C., Zhou, R., Tang, Z. and Li, K. (2015) Wnt antagonist, secreted frizzled-related protein 1, is involved in prenatal skeletal muscle development and is a target of miRNA-1/206 in pigs. *BMC Mol Biol*, **16**, 4.
19. Sun, J., Sonstegard, T.S., Li, C., Huang, Y., Li, Z., Lan, X., Zhang, C., Lei, C., Zhao, X. and Chen, H. (2015) Altered microRNA expression in bovine skeletal muscle with age. *Anim Genet*, [Epub ahead of print].
20. Bhattacharjya, S., Roy, K.S., Ganguly, A., Sarkar, S., Panda, C.K., Bhattacharyya, D., Bhattacharyya, N.P. and Roychoudhury, S. (2015) Inhibition of nucleoporin member Nup214 expression by miR-133b perturbs mitotic timing and leads to cell death. *Mol Cancer*, **14**, 42.
21. Miao, C.G., Shi, W.J., Xiong, Y.Y., Yu, H., Zhang, X.L., Qin, M.S., Du, C.L., Song, T.W. and Li, J. (2015) miR-375 regulates the canonical Wnt pathway through FZD8 silencing in arthritis synovial fibroblasts. *Immunol Lett*, **164**, 1-10.
22. He, S., Zeng, S., Zhou, Z.W., He, Z.X. and Zhou, S.F. (2015) Hsa-microRNA-181a is a regulator of a number of cancer genes and a biomarker for endometrial carcinoma in patients: a bioinformatic and clinical study and the therapeutic implication. *Drug Des Devel Ther*, **9**, 1103-1175.
23. Tian, R., Wang, R.L., Xie, H., Jin, W. and Yu, K.L. (2013) Overexpressed miRNA-155 dysregulates intestinal epithelial apical junctional complex in severe acute pancreatitis. *World J Gastroenterol*, **19**, 8282-8291.
24. Ma, Y.J., Yang, J., Fan, X.L., Zhao, H.B., Hu, W., Li, Z.P., Yu, G.C., Ding, X.R., Wang, J.Z., Bo, X.C. *et al.* (2012) Cellular microRNA let-7c inhibits M1 protein expression of the H1N1 influenza A virus in infected human lung epithelial cells. *J Cell Mol Med*, **16**, 2539-2546.
25. Xin, F., Li, M., Balch, C., Thomson, M., Fan, M., Liu, Y., Hammond, S.M., Kim, S. and Nephew, K.P. (2009) Computational analysis of microRNA profiles and their target

genes suggests significant involvement in breast cancer antiestrogen resistance. *Bioinformatics*, **25**, 430-434.

26. Zhang, X., Ni, Z., Duan, Z., Xin, Z., Wang, H., Tan, J., Wang, G. and Li, F. (2015) Overexpression of E2F mRNAs associated with gastric cancer progression identified by the transcription factor and miRNA co-regulatory network analysis. *PLoS One*, **10**, e0116979.
27. Sevinc, E.D., Egeli, U., Cecener, G., Tezcan, G., Tunca, B., Gokgoz, S., Tasdelen, I., Tolunay, S. and Evrensel, T. (2015) Association of miR-1266 with recurrence/metastasis potential in estrogen receptor positive breast cancer patients. *Asian Pac J Cancer Prev*, **16**, 291-297.
28. Long, X.H., Mao, J.H., Peng, A.F., Zhou, Y., Huang, S.H. and Liu, Z.L. (2013) Tumor suppressive microRNA-424 inhibits osteosarcoma cell migration and invasion via targeting fatty acid synthase. *Exp Ther Med*, **5**, 1048-1052.
29. Yan, H., Dong, X., Zhong, X., Ye, J., Zhou, Y., Yang, X., Shen, J. and Zhang, J. (2014) Inhibitions of epithelial to mesenchymal transition and cancer stem cells-like properties are involved in miR-148a-mediated anti-metastasis of hepatocellular carcinoma. *Mol Carcinog*, **53**, 960-969.
30. Eichelser, C., Stuckrath, I., Muller, V., Milde-Langosch, K., Wikman, H., Pantel, K. and Schwarzenbach, H. (2014) Increased serum levels of circulating exosomal microRNA-373 in receptor-negative breast cancer patients. *Oncotarget*, **5**, 9650-9663.
31. Guduric-Fuchs, J., O'Connor, A., Camp, B., O'Neill, C.L., Medina, R.J. and Simpson, D.A. (2012) Selective extracellular vesicle-mediated export of an overlapping set of microRNAs from multiple cell types. *BMC Genomics*, **13**, 357.
32. Kluiver, J., Gibcus, J.H., Hettinga, C., Adema, A., Richter, M.K., Halsema, N., Slezak-Prochazka, I., Ding, Y., Kroesen, B.J. and van den Berg, A. (2012) Rapid generation of microRNA sponges for microRNA inhibition. *PLoS One*, **7**, e29275.
33. Malhas, A., Saunders, N.J. and Vaux, D.J. (2010) The nuclear envelope can control gene expression and cell cycle progression via miRNA regulation. *Cell Cycle*, **9**, 531-539.
34. Kotlabova, K., Doucha, J. and Hromadnikova, I. (2011) Placental-specific microRNA in maternal circulation--identification of appropriate pregnancy-associated microRNAs with diagnostic potential. *J Reprod Immunol*, **89**, 185-191.
35. Kogure, T., Lin, W.L., Yan, I.K., Braconi, C. and Patel, T. (2011) Intercellular nanovesicle-mediated microRNA transfer: a mechanism of environmental modulation of hepatocellular cancer cell growth. *Hepatology*, **54**, 1237-1248.
36. Pallocca, G., Fabbri, M., Sacco, M.G., Gribaldo, L., Pamies, D., Laurenza, I. and Bal-Price, A. (2013) miRNA expression profiling in a human stem cell-based model as a tool for developmental neurotoxicity testing. *Cell Biol Toxicol*, **29**, 239-257.
37. Ninomiya, M., Kondo, Y., Funayama, R., Nagashima, T., Kogure, T., Kakazu, E., Kimura, O., Ueno, Y., Nakayama, K. and Shimosegawa, T. (2013) Distinct microRNAs expression profile in primary biliary cirrhosis and evaluation of miR 505-3p and miR197-3p as novel biomarkers. *PLoS One*, **8**, e66086.
38. Gan, L., Schwengberg, S. and Denecke, B. (2011) MicroRNA profiling during cardiomyocyte-specific differentiation of murine embryonic stem cells based on two different miRNA array platforms. *PLoS One*, **6**, e25809.
39. Lien, G.S., Liu, J.F., Chien, M.H., Hsu, W.T., Chang, T.H., Ku, C.C., Ji, A.T., Tan, P., Hsieh, T.L., Lee, L.M. *et al.* (2014) The ability to suppress macrophage-mediated

inflammation in orbital fat stem cells is controlled by miR-671-5p. *Stem Cell Res Ther*, **5**, 97.

40. Braga, E.A., Loginov, V.I., Pronina, I.V., Khodyrev, D.S., Rykov, S.V., Burdennyy, A.M., Friedman, M.V., Kazubskaya, T.P., Kubatiev, A.A. and Kushlinskii, N.E. (2015) Upregulation of RHOA and NKIRAS1 Genes in Lung Tumors Is Associated with Loss of Their Methylation as well as with Methylation of Regulatory miRNA Genes. *Biochemistry (Mosc)*, **80**, 483-494.
41. Lin, L., Zheng, Y., Tu, Y., Wang, Z., Liu, H., Lu, X., Xu, L. and Yuan, J. (2015) MicroRNA-144 suppresses tumorigenesis and tumor progression of astrocytoma by targeting EZH2. *Hum Pathol*, [Epub ahead of print].
42. Romero-Cordoba, S., Rodriguez-Cuevas, S., Rebollar-Vega, R., Quintanar-Jurado, V., Maffuz-Aziz, A., Jimenez-Sanchez, G., Bautista-Pina, V., Arellano-Llamas, R. and Hidalgo-Miranda, A. (2012) Identification and pathway analysis of microRNAs with no previous involvement in breast cancer. *PLoS One*, **7**, e31904.
43. Pio, G., Ceci, M., D'Elia, D., Loglisci, C. and Malerba, D. (2013) A novel biclustering algorithm for the discovery of meaningful biological correlations between microRNAs and their target genes. *BMC Bioinformatics*, **14 Suppl 7**, S8.
44. Edinger, R.S., Coronnello, C., Bodnar, A.J., LaFramboise, W.A., Benos, P.V., Ho, J., Johnson, J.P. and Butterworth, M.B. (2014) Aldosterone regulates microRNAs in the cortical collecting duct to alter sodium transport. *J Am Soc Nephrol*, **25**, 2445-2457.
45. Li, J., Chen, Z., Zhao, J., Fang, L., Fang, R., Xiao, J., Chen, X., Zhou, A., Zhang, Y., Ren, L. *et al.* (2015) Difference in microRNA expression and editing profile of lung tissues from different pig breeds related to immune responses to HP-PRRSV. *Sci Rep*, **5**, 9549.
46. Desjardin, C., Vaiman, A., Mata, X., Legendre, R., Laubier, J., Kennedy, S.P., Laloe, D., Barrey, E., Jacques, C., Cribiu, E.P. *et al.* (2014) Next-generation sequencing identifies equine cartilage and subchondral bone miRNAs and suggests their involvement in osteochondrosis physiopathology. *BMC Genomics*, **15**, 798.
47. Canturk, K.M., Ozdemir, M., Can, C., Oner, S., Emre, R., Aslan, H., Cilingir, O., Ciftci, E., Celayir, F.M., Aldemir, O. *et al.* (2014) Investigation of key miRNAs and target genes in bladder cancer using miRNA profiling and bioinformatic tools. *Mol Biol Rep*, **41**, 8127-8135.
48. Dymacek, J., Snyder-Talkington, B.N., Porter, D.W., Mercer, R.R., Wolfarth, M.G., Castranova, V., Qian, Y. and Guo, N.L. (2015) mRNA and miRNA regulatory networks reflective of multi-walled carbon nanotube-induced lung inflammatory and fibrotic pathologies in mice. *Toxicol Sci*, **144**, 51-64.
49. Kou, Y., Qiao, L. and Wang, Q. (2015) Identification of core miRNA based on small RNA-seq and RNA-seq for colorectal cancer by bioinformatics. *Tumour Biol*, **36**, 2249-2255.
50. Ouyang, M., Li, Y., Ye, S., Ma, J., Lu, L., Lv, W., Chang, G., Li, X., Li, Q., Wang, S. *et al.* (2014) MicroRNA profiling implies new markers of chemoresistance of triple-negative breast cancer. *PLoS One*, **9**, e96228.
51. Zaragosi, L.E., Wdziekonski, B., Brigand, K.L., Villageois, P., Mari, B., Waldmann, R., Dani, C. and Barbry, P. (2011) Small RNA sequencing reveals miR-642a-3p as a novel adipocyte-specific microRNA and miR-30 as a key regulator of human adipogenesis. *Genome Biol*, **12**, R64.

52. Lino Cardenas, C.L., Henaoui, I.S., Courcot, E., Roderburg, C., Cauffiez, C., Aubert, S., Copin, M.C., Wallaert, B., Glowacki, F., Dewaeles, E. *et al.* (2013) miR-199a-5p Is upregulated during fibrogenic response to tissue injury and mediates TGFbeta-induced lung fibroblast activation by targeting caveolin-1. *PLoS Genet*, **9**, e1003291.
53. Maegdefessel, L., Spin, J.M., Raaz, U., Eken, S.M., Toh, R., Azuma, J., Adam, M., Nagakami, F., Heymann, H.M., Chernugobova, E. *et al.* (2014) miR-24 limits aortic vascular inflammation and murine abdominal aneurysm development. *Nat Commun*, **5**, 5214.
54. Cohen, J.E., Lee, P.R. and Fields, R.D. (2014) Systematic identification of 3'-UTR regulatory elements in activity-dependent mRNA stability in hippocampal neurons. *Philos Trans R Soc Lond B Biol Sci*, **369**.
55. Tan, G.C., Chan, E., Molnar, A., Sarkar, R., Alexieva, D., Isa, I.M., Robinson, S., Zhang, S., Ellis, P., Langford, C.F. *et al.* (2014) 5' isomiR variation is of functional and evolutionary importance. *Nucleic Acids Res*, **42**, 9424-9435.
56. Bruni, R., Marcantonio, C., Tritarelli, E., Tataseo, P., Stellacci, E., Costantino, A., Villano, U., Battistini, A. and Ciccaglione, A.R. (2011) An integrated approach identifies IFN-regulated microRNAs and targeted mRNAs modulated by different HCV replicon clones. *BMC Genomics*, **12**, 485.
57. Naderi, E., Mostafaei, M., Pourshams, A. and Mohamadkhani, A. (2014) Network of microRNAs-mRNAs interactions in pancreatic cancer. *Biomed Res Int*, **2014**, 534821.
58. Hiddingh, L., Raktoe, R.S., Jeuken, J., Hulleman, E., Noske, D.P., Kaspers, G.J., Vandertop, W.P., Wesseling, P. and Wurdinger, T. (2014) Identification of temozolomide resistance factors in glioblastoma via integrative miRNA/mRNA regulatory network analysis. *Sci Rep*, **4**, 5260.
59. Coppola, A., Romito, A., Borel, C., Gehrig, C., Gagnebin, M., Falconnet, E., Izzo, A., Altucci, L., Banfi, S., Antonarakis, S.E. *et al.* (2014) Cardiomyogenesis is controlled by the miR-99a/let-7c cluster and epigenetic modifications. *Stem Cell Res*, **12**, 323-337.
60. Hafner, M., Landthaler, M., Burger, L., Khorshid, M., Hausser, J., Berninger, P., Rothballer, A., Ascano, M., Jr., Jungkamp, A.C., Munschauer, M. *et al.* (2010) Transcriptome-wide identification of RNA-binding protein and microRNA target sites by PAR-CLIP. *Cell*, **141**, 129-141.
61. Bostjancic, E., Zidar, N. and Glavac, D. (2012) MicroRNAs and cardiac sarcoplasmic reticulum calcium ATPase-2 in human myocardial infarction: expression and bioinformatic analysis. *BMC Genomics*, **13**, 552.
62. Vinnikov, I.A., Hajdukiewicz, K., Reymann, J., Beneke, J., Czajkowski, R., Roth, L.C., Novak, M., Roller, A., Dorner, N., Starkuviene, V. *et al.* (2014) Hypothalamic miR-103 protects from hyperphagic obesity in mice. *J Neurosci*, **34**, 10659-10674.
63. Ganesan, J., Ramanujam, D., Sassi, Y., Ahles, A., Jentzsch, C., Werfel, S., Leierseder, S., Loyer, X., Giacca, M., Zentilin, L. *et al.* (2013) MiR-378 controls cardiac hypertrophy by combined repression of mitogen-activated protein kinase pathway factors. *Circulation*, **127**, 2097-2106.
64. Liu, Y.J., Lin, Y.F., Chen, Y.F., Luo, E.C., Sher, Y.P., Tsai, M.H., Chuang, E.Y. and Lai, L.C. (2013) MicroRNA-449a enhances radiosensitivity in CL1-0 lung adenocarcinoma cells. *PLoS One*, **8**, e62383.
65. Levanen, B., Bhakta, N.R., Torregrosa Paredes, P., Barbeau, R., Hiltbrunner, S., Pollack, J.L., Skold, C.M., Svartengren, M., Grunewald, J., Gabrielsson, S. *et al.* (2013) Altered

microRNA profiles in bronchoalveolar lavage fluid exosomes in asthmatic patients. *J Allergy Clin Immunol*, **131**, 894-903.
66. Zovoilis, A., Agbemenyah, H.Y., Agis-Balboa, R.C., Stilling, R.M., Edbauer, D., Rao, P., Farinelli, L., Delalle, I., Schmitt, A., Falkai, P. *et al.* (2011) microRNA-34c is a novel target to treat dementias. *Embo J*, **30**, 4299-4308.
67. Volinia, S., Galasso, M., Costinean, S., Tagliavini, L., Gamberoni, G., Drusco, A., Marchesini, J., Mascellani, N., Sana, M.E., Abu Jarour, R. *et al.* (2010) Reprogramming of miRNA networks in cancer and leukemia. *Genome Res*, **20**, 589-599.
68. Li, B., Lu, Q., Song, Z.G., Yang, L., Jin, H., Li, Z.G., Zhao, T.J., Bai, Y.F., Zhu, J., Chen, H.Z. *et al.* (2013) Functional analysis of DNA methylation in lung cancer. *Eur Rev Med Pharmacol Sci*, **17**, 1191-1197.
69. Hsu, S.D., Huang, H.Y., Chou, C.H., Sun, Y.M., Hsu, M.T. and Tsou, A.P. (2015) Integrated analyses to reconstruct microRNA-mediated regulatory networks in mouse liver using high-throughput profiling. *BMC Genomics*, **16 Suppl 2**, S12.
70. Lai, X., Schmitz, U., Gupta, S.K., Bhattacharya, A., Kunz, M., Wolkenhauer, O. and Vera, J. (2012) Computational analysis of target hub gene repression regulated by multiple and cooperative miRNAs. *Nucleic Acids Res*, **40**, 8818-8834.
71. Barh, D., Jain, N., Tiwari, S., Field, J.K., Padin-Iruegas, E., Ruibal, A., Lopez, R., Herranz, M., Bhattacharya, A., Juneja, L. *et al.* (2013) A novel in silico reverse-transcriptomics-based identification and blood-based validation of a panel of sub-type specific biomarkers in lung cancer. *BMC Genomics*, **14 Suppl 6**, S5.
72. Delfino, K.R. and Rodriguez-Zas, S.L. (2013) Transcription factor-microRNA-target gene networks associated with ovarian cancer survival and recurrence. *PLoS One*, **8**, e58608.
73. Chan, L.W. (2011) Modeling equilibrium of microRNA expression. *Front Genet*, **2**, 35.
74. Liu, Z., Borlak, J. and Tong, W. (2014) Deciphering miRNA transcription factor feed-forward loops to identify drug repurposing candidates for cystic fibrosis. *Genome Med*, **6**, 94.
75. Angerstein, C., Hecker, M., Paap, B.K., Koczan, D., Thamilarasan, M., Thiesen, H.J. and Zettl, U.K. (2012) Integration of MicroRNA databases to study MicroRNAs associated with multiple sclerosis. *Mol Neurobiol*, **45**, 520-535.
76. Madhavan, S., Gusev, Y., Natarajan, T.G., Song, L., Bhuvaneshwar, K., Gauba, R., Pandey, A., Haddad, B.R., Goerlitz, D., Cheema, A.K. *et al.* (2013) Genome-wide multi-omics profiling of colorectal cancer identifies immune determinants strongly associated with relapse. *Front Genet*, **4**, 236.
77. Sun, J., Gong, X., Purow, B. and Zhao, Z. (2012) Uncovering MicroRNA and Transcription Factor Mediated Regulatory Networks in Glioblastoma. *PLoS Comput Biol*, **8**, e1002488.
78. Wu, X. and Song, Y. (2011) Preferential regulation of miRNA targets by environmental chemicals in the human genome. *BMC Genomics*, **12**, 244.
79. Chen, C.J., Cox, J.E., Azarm, K.D., Wylie, K.N., Woolard, K.D., Pesavento, P.A. and Sullivan, C.S. (2015) Identification of a polyomavirus microRNA highly expressed in tumors. *Virology*, **476**, 43-53.
80. Kaminski, M.J., Kaminska, M., Skorupa, I., Kazimierczyk, R., Musial, W.J. and Kaminski, K.A. (2013) In-silico identification of cardiovascular disease-related SNPs affecting predicted microRNA target sites. *Pol Arch Med Wewn*, **123**, 355-363.

81. Li, Y., Li, Z., Zhou, S., Wen, J., Geng, B., Yang, J. and Cui, Q. (2013) Genome-wide analysis of human microRNA stability. *Biomed Res Int*, **2013**, 368975.
82. He, N., Zheng, H., Li, P., Zhao, Y., Zhang, W., Song, F. and Chen, K. (2014) miR-485-5p binding site SNP rs8752 in HPGD gene is associated with breast cancer risk. *PLoS One*, **9**, e102093.
83. Rushefski, M., Aplenc, R., Meyer, N., Li, M., Feng, R., Lanken, P.N., Gallop, R., Bellamy, S., Localio, A.R., Feinstein, S.I. *et al.* (2011) Novel variants in the PRDX6 Gene and the risk of Acute Lung Injury following major trauma. *BMC Med Genet*, **12**, 77.
84. Bridges, T.M., Scheraga, R.G., Tulapurkar, M.E., Suffredini, D., Liggett, S.B., Ramarathnam, A., Potla, R., Singh, I.S. and Hasday, J.D. (2015) Polymorphisms in human heat shock factor-1 and analysis of potential biological consequences. *Cell Stress Chaperones*, **20**, 47-59.
85. Paolicchi, E., Pacetti, P., Giovannetti, E., Mambrini, A., Orlandi, M., Crea, F., Romani, A.A., Tartarini, R., Danesi, R., Peters, G.J. *et al.* (2013) A single nucleotide polymorphism in EZH2 predicts overall survival rate in patients with cholangiocarcinoma. *Oncol Lett*, **6**, 1487-1491.
86. Lu, S., Bevier, M., Huhn, S., Sainz, J., Lascorz, J., Pardini, B., Naccarati, A., Vodickova, L., Novotny, J., Hemminki, K. *et al.* (2013) Genetic variants in C-type lectin genes are associated with colorectal cancer susceptibility and clinical outcome. *Int J Cancer*, **133**, 2325-2333.
87. Pardini, B., Rosa, F., Barone, E., Di Gaetano, C., Slyskova, J., Novotny, J., Levy, M., Garritano, S., Vodickova, L., Buchler, T. *et al.* (2013) Variation within 3'-UTRs of base excision repair genes and response to therapy in colorectal cancer patients: A potential modulation of microRNAs binding. *Clin Cancer Res*, **19**, 6044-6056.
88. Vaishnavi, V., Manikandan, M. and Munirajan, A.K. (2014) Mining the 3'UTR of autism-implicated genes for SNPs perturbing microRNA regulation. *Genomics Proteomics Bioinformatics*, **12**, 92-104.
89. Song, C.Q., Zhang, J.H., Shi, J.C., Cao, X.Q., Song, C.H., Hassan, A., Wang, P., Dai, L.P., Zhang, J.Y. and Wang, K.J. (2014) Bioinformatic prediction of SNPs within miRNA binding sites of inflammatory genes associated with gastric cancer. *Asian Pac J Cancer Prev*, **15**, 937-943.
90. Boudreau, R.L., Jiang, P., Gilmore, B.L., Spengler, R.M., Tirabassi, R., Nelson, J.A., Ross, C.A., Xing, Y. and Davidson, B.L. (2014) Transcriptome-wide discovery of microRNA binding sites in human brain. *Neuron*, **81**, 294-305.
91. Chen, X., Zang, W., Xue, F., Shen, Z. and Zhang, Q. (2013) Bioinformatics analysis reveals potential candidate drugs for different subtypes of glioma. *Neurol Sci*, **34**, 1139-1143.
92. Jia, M., Yang, B., Li, Z., Shen, H., Song, X. and Gu, W. (2014) Computational analysis of functional single nucleotide polymorphisms associated with the CYP11B2 gene. *PLoS One*, **9**, e104311.
93. Li, M.J. and Wang, J. (2015) Current trend of annotating single nucleotide variation in humans-A case study on SNVrap. *Methods*, **79**, 32-40.
94. Murakami, Y., Aly, H.H., Tajima, A., Inoue, I. and Shimotohno, K. (2009) Regulation of the hepatitis C virus genome replication by miR-199a. *J Hepatol*, **50**, 453-460.

95. Wu, Y.L., Wu, C.P., Liu, C.Y., Hsu, P.W., Wu, E.C. and Chao, Y.C. (2011) A non-coding RNA of insect HzNV-1 virus establishes latent viral infection through microRNA. *Sci Rep*, **1**, 60.
96. Veksler-Lublinsky, I., Shemer-Avni, Y., Meiri, E., Bentwich, Z., Kedem, K. and Ziv-Ukelson, M. (2012) Finding quasi-modules of human and viral miRNAs: a case study of human cytomegalovirus (HCMV). *BMC Bioinformatics*, **13**, 322.
97. Elefant, N., Altuvia, Y. and Margalit, H. (2011) A wide repertoire of miRNA binding sites: prediction and functional implications. *Bioinformatics*, **27**, 3093-3101.
