# Supplementary material for: Bioinformatic tools for microRNA dissection
Source: Nucleic Acids Res. 2015 Nov 17;44(1):24–44. doi: 10.1093/nar/gkv1221 (PMC4705652; doi:10.1093/nar/gkv1221)
Supplement: SUPPLEMENTARY DATA [file supp_gkv1221_nar-03711-survey-d-2014-File007.pdf]

Supporting material S3: Bioinformatic resources of microRNA (miRNA)

Category	Features	Tools	URL	References
**MiRNA Target prediction**	Target site prediction	Microinspector	http://bioinfo1.uni-plovdiv.bg/cgi-bin/microinspector/	(1)
	Target predictions	miTarget	http://www.webcitation.org/query.php?url=http://cbit.snu.ac.kr/~miTarget&refdoi=10.1186/1471-2105-7-411	(2)
	Target prediction	MicroTar	http://tiger.dbs.nus.edu.sg/microtar/	(3)
	Naïve Bayes for miRNA target predictions, a machine learning approach	NBmiRTar	http://wotan.wistar.upenn.edu/NBmiRTar/login.php	(4)
	Integrative human miRNA target prediction	ExprTarget	http://www.scandb.org/apps/microrna/index.html	(5)
	Target prediction	TargetRank	http://hollywood.mit.edu/targetrank/	(6)
	Human miRNA targets prediction by sequence-based method	HuMiTar	Not found	(7)
	SVM based algorithm that serves as a post-processing filter for the miRNA: target duplexes predicted by softwares such as miRanda, PicTar and TargetScanS.	MirTif	http://mirtif.bii.a-star.edu.sg/	(8)
	MiRNA target prediction with systematic identification of tissue-specific negative examples	TargetMiner	www.isical.ac.in/~bioinfo_miu	(9)
	This tool uses computational target predictions in order to automatically infer the processes affected by human miRNAs.	FAME	http://acgt.cs.tau.ac.il/fame/	(10)
	MiRNA target prediction by supervised machine learning approach	TargetSpy	http://www.targetspy.org/	(11)
	A database for miRNA target predictions in coding and	miRNA_Targets	http://mamsap.it.deakin.edu.au/mirna_targets/	(12)

	non-coding regions of mRNAs			
	An algorithm of miRNA target prediction in *Homo sapiens*	HomoTarget	http://lbb.ut.ac.ir/Download/LBBsoft/homoTarget/	(13)
	Predicts miRNA-mRNA interactions based on relative ranking of matched expression data	miMsg	http://www.martinrijlaarsdam.nl/mimsg	(14)
	Predicts human miRNA target genes with a random forest classifier	RFMirTarget	Not found	(15)
	A two-step dynamic interaction model that accounts for mRNA accessibility and Pumilio binding accurately predicts miRNA targets	MREdictor	http://mredictor.hugef-research.org/	(16)
	Target prediction	MicroCosm Targets Version 5	http://www.ebi.ac.uk/enright-srv/microcosm/htdocs/targets/v5/	Not found
	Target site prediction	MAMI	http://mami.med.harvard.edu/	Not found
	A curated database of human, mouseand rat miRNA–mRNA targets	miRGate	http://mirgate.bioinfo.cnio.es/API/	(17)
	Graphics processing units based miRNA target analysis through CUDA-miRanda	GAMUT	https://sourceforge.net/projects/cudamiranda/	(18)
**MiRNA finding**	Classify the real and pseudo miRNA precursors using random forest prediction model with combined features	MiPred	http://www.bioinf.seu.edu.cn/miRNA/	(19)
	Web-based tool used for homologous miRNA gene search in several species	miRNAminer	http://groups.csail.mit.edu/pag/mirnaminer/	(20)
	A web server for identification of miRNA precursors in a given DNA sequence, utilizing secondary structure-based filtering systems and an algorithm based on stochastic context free grammar trained on human miRNAs.	CID-miRNA	http://mirna.jnu.ac.in/cidmirna/	(21)
	A database facilitates the	deepBase	http://deepbase.sysu.edu.cn/	(22)

	comprehensive annotation and discovery of small RNAs from transcriptomic data			
	A database presents the results of a comprehensive computational survey of miRNA gene candidates across the majority of sequenced metazoan genomes.	miROrtho	http://cegg.unige.ch/mirortho	(23)
	Provides detailed annotation information for known miRNAs, such as miRNA/miRNA*, predicts novel miRNAs that have not been characterized before.	mirTools	http://centre.bioinformatics.zj.cn/mirtools/	(24)
	A tool designed to locate miRNA precursor sequences in existing genomic sequences using potential mature miRNA sequences as input	MapMi	http://www.ebi.ac.uk/enright-srv/MapMi/	(25)
	A computational method for the systematic identification of miRNAs from high throughput sequencing data	miRTRAP	http://flybuzz.berkeley.edu/miRTRAP.html	(26)
	A pre-miRNA gene prediction algorithm in Bos taurus	BosFinder	http://lbb.ut.ac.ir/Download/LBBsoft/BosFinder/	(27)
	An integrative approach in miRNA search and annotation	miRNEST	http://lemur.amu.edu.pl/share/php/mirnest/	(28,29)
**Correlating miRNA and mRNA expression**	A database containing expression information for human, mouse, rat, zebrafish, worm and fruitfly small RNAs (mostly miRNAs)	smirnaDB	http://www.mirz.unibas.ch/cloningprofiles/	(30)
	Combines results of other miRNA target prediction programs with paired miRNA-mRNA expression profiling	GenMiR++	http://www.psi.toronto.edu/genmir/	(31)
	An integrated database of	FANTOM4	http://fantom.gsc.riken.jp/4/edgeexpress/view/#5	(32)

promoters, genes, miRNAs, expression dynamics and regulatory interactions	EdgeExpressDB	558263	
An integrated miRNA expression atlasand target prediction resource	MirZ	http://www.mirz.unibas.ch/	(33)
A systematic method, for extracting miRNA expression profiles from sequencing reads generated by second-generation sequencing	miRExpress	http://mirexpress.mbc.nctu.edu.tw/	(34)
Web tool for examining biological functions of miRNA expression	MMIA	http://cancer.informatics.indiana.edu/mmia	(35)
A miRNA expression atlas of the mouse eye	miRNeye	http://mirneye.tigem.it/	(36)
A miRNA expression profiler and classification resource designed to identify functional correlations between miRNAs and their targets	mimiRNA	http://mimirna.centenary.org.au/	(37)
Web-server for automated detection of miRNA effects from expression data	SylArray	http://www.ebi.ac.uk/enright-srv/sylarray/	(38)
Sarcoma/colon cancer miRNA expression database	S-MED/cc-MED	http://www.oncomir.umn.edu/	(39,40)
This tool is based on integration of expression profiling and sequence-based miRNA target recognition softwares.	HOCTAR	http://hoctar.tigem.it/	(41)
This approach is based on a multi-level integration of corresponding miRNA and mRNA gene expression levels, miRNA target prediction, transcription factor target prediction and mechanistic models of gene network regulation.	The microRNA body map	http://www.mirnabodymap.org/	(42)
A resource for predicted miRNA targets and expression	Rfam 11.0	http://rfam.sanger.ac.uk/	(43)

**Others**	A suite of tools for analysing and visualizing next generation sequencing miRNA and small RNA datasets	The UEA sRNA workbench	http://srna-workbench.cmp.uea.ac.uk/	(44)
	Scores based on similarity to 50 pairs of experimentally verified miRNA hairpins	miRScan	http://genes.mit.edu/mirscan/	(45,46)
	Classify real and pseudo miRNA precursors using local structure-sequence features and support vector machine	Triplet-SVM	http://bioinfo.au.tsinghua.edu.cn/mirnasvm/	(47)
	A database for the study of animal miRNA genomic organization and function	miRGen	http://diana.cslab.ece.ntua.gr/mirgen/	(48,49)
	Identifies signature of miRNA-regulatory activity in given gene list	GeneSet2MiRNA	http://mips.helmholtz-muenchen.de/proj/gene2mir/	(50)
	A unified programming interface for miRNA data resources	miRMaid	http://current.mirmaid.org/	(51)
	A method for enrichment and depletion analysis of a miRNA category in a list of miRNAs	TAM	http://202.38.126.151/hmdd/tools/tam.html/	(52)
	Automated extraction of associations between miRNAs and genes from the biomedical literature	miRSel	http://services.bio.ifi.lmu.de/mirsel/	(53)
	A comprehensive database for genomic variations in miRNAs	miRvar	http://genome.igib.res.in/mirlovd/home.php	(54)
	A multi-species miRNA homologous viewer	miRviewer	http://people.csail.mit.edu/akiezun/microRNAviewer/index.html	(55)
	Performs survival analyses for the input miRNA across multiple expression datasets	MIRUMIR	http://www.bioprofiling.de/GEO/MIRUMIR/mirumir.html	(56)

An experimentally supported database of mRNA deregulation in various cancers	TUMIR	http://www.ncrnalab.com/TUMIR/	(57)
A SVM based method for predicting human Dicer cleavage sites using sequence and secondary structure of miRNA precursors	PHDcleav	http://www.imtech.res.in/raghava/phdcleav/	(58)
MIROR: a method for cell-type specific miRNA occupancy rate prediction	MIROR	http://bioinfo.au.tsinghua.edu.cn/member/xwwang/MIROR/	(59)

REFERENCES

1. Rusinov, V., Baev, V., Minkov, I.N. and Tabler, M. (2005) MicroInspector: a web tool for detection of miRNA binding sites in an RNA sequence. *Nucleic acids research*, **33**, W696-W700.

2. Kim, S.-K., Nam, J.-W., Rhee, J.-K., Lee, W.-J. and Zhang, B.-T. (2006) miTarget: microRNA target gene prediction using a support vector machine. *BMC bioinformatics*, **7**, 411.

3. Thadani, R. and Tammi, M.T. (2006) MicroTar: predicting microRNA targets from RNA duplexes. *BMC bioinformatics*, **7**, S20.

4. Yousef, M., Jung, S., Kossenkov, A.V., Showe, L.C. and Showe, M.K. (2007) Naïve Bayes for microRNA target predictions-machine learning for microRNA targets. *Bioinformatics*, **23**, 2987-2992.

5. Gamazon, E.R., Im, H.-K., Duan, S., Lussier, Y.A., Cox, N.J., Dolan, M.E. and Zhang, W. (2010) Exprtarget: an integrative approach to predicting human microRNA targets. *PLoS One*, **5**, e13534.

6. Nielsen, C.B., Shomron, N., Sandberg, R., Hornstein, E., Kitzman, J. and Burge, C.B. (2007) Determinants of targeting by endogenous and exogenous microRNAs and siRNAs. *Rna*, **13**, 1894-1910.

7. Ruan, J., Chen, H., Kurgan, L., Chen, K., Kang, C. and Pu, P. (2008) HuMiTar: a sequence-based method for prediction of human microRNA targets. *Algorithms Mol. Biol*, **3**, 16.

8. Yang, Y., Wang, Y.-P. and Li, K.-B. (2008) MiRTif: a support vector machine-based microRNA target interaction filter. *BMC bioinformatics*, **9**, S4.

9. Bandyopadhyay, S. and Mitra, R. (2009) TargetMiner: microRNA target prediction with systematic identification of tissue-specific negative examples. *Bioinformatics*, **25**, 2625-2631.

10. Ulitsky, I., Laurent, L.C. and Shamir, R. (2010) Towards computational prediction of microRNA function and activity. *Nucleic acids research*, **38**, e160-e160.

11. Sturm, M., Hackenberg, M., Langenberger, D. and Frishman, D. (2010) TargetSpy: a supervised machine learning approach for microRNA target prediction. *BMC bioinformatics*, **11**, 292.

12. Kumar, A., Wong, A.K.L., Tizard, M.L., Moore, R.J. and Lefèvre, C. (2012) miRNA_Targets: A database for miRNA target predictions in coding and non-coding regions of mRNAs. *Genomics*, **100**, 352-356.

13. Ahmadi, H., Ahmadi, A., Azimzadeh-Jamalkandi, S., Shoorehdeli, M.A., Salehzadeh-Yazdi, A., Bidkhori, G. and Masoudi-Nejad, A. (2013) HomoTarget: A new algorithm for prediction of microRNA targets in *Homo sapiens*. *Genomics*, **101**, 94-100.

14. Rijlaarsdam, M.A., Rijlaarsdam, D.J., Gillis, A.J.M., Dorssers, L.C.J. and Looijenga, L.H.J. (2013) miMsg: a target enrichment algorithm for predicted miR-mRNA interactions based on relative ranking of matched expression data. *Bioinformatics*, btt246.

15. Mendoza, M.R., da Fonseca, G.C., Loss-Morais, G., Alves, R., Margis, R. and Bazzan, A.L.C. (2013) RFMirTarget: predicting human microRNA target genes with a random forest classifier. *PloS one*, **8**, e70153.

16. Incarnato, D., Neri, F., Diamanti, D. and Oliviero, S. (2013) MREdictor: a two-step dynamic interaction model that accounts for mRNA accessibility and Pumilio binding accurately predicts microRNA targets. *Nucleic acids research*, gkt629.

17. Andrés-León, E., Pen˜a, D.G.l., Go´mez-Lo´pez, G. and Pisano, D.G. (2015) miRGate: a curated database of human, mouse and rat miRNA-mRNA targets. *Database*, **bav035**, 1-9.

18. Wang, S., Kim, J., Jiang, X., Brunner, S.F. and Ohno-Machado, L. (2014) GAMUT: GPU accelerated microRNA analysis to uncover target genes through CUDA-miRanda. *BMC Medical Genomics*, **7**, 1-13.

19. Jiang, P., Wu, H., Wang, W., Ma, W., Sun, X. and Lu, Z. (2007) MiPred: classification of real and pseudo microRNA precursors using random forest prediction model with combined features. *Nucleic acids research*, **35**, W339-W344.

20. Artzi, S., Kiezun, A. and Shomron, N. (2008) miRNAminer: a tool for homologous microRNA gene search. *BMC bioinformatics*, **9**, 39.

21. Tyagi, S., Vaz, C., Gupta, V., Bhatia, R., Maheshwari, S., Srinivasan, A. and Bhattacharya, A. (2008) CID-miRNA: a web server for prediction of novel miRNA precursors in human genome. *Biochemical and biophysical research communications*, **372**, 831-834.

22. Yang, J.-H., Shao, P., Zhou, H., Chen, Y.-Q. and Qu, L.-H. (2009) deepBase: a database for deeply annotating and mining deep sequencing data. *Nucleic acids research*, gkp943.

23. Gerlach, D., Kriventseva, E.V., Rahman, N., Vejnar, C.E. and Zdobnov, E.M. (2009) miROrtho: computational survey of microRNA genes. *Nucleic acids research*, **37**, D111-D117.

24. Zhu, E., Zhao, F., Xu, G., Hou, H., Zhou, L., Li, X., Sun, Z. and Wu, J. (2010) mirTools: microRNA profiling and discovery based on high-throughput sequencing. *Nucleic acids research*, **38**, W392-W397.

25. Guerra-Assunção, J.A. and Enright, A.J. (2010) MapMi: automated mapping of microRNA loci. *BMC bioinformatics*, **11**, 133.

26. Hendrix, D., Levine, M. and Shi, W. (2010) Method miRTRAP, a computational method for the systematic identification of miRNAs from high throughput sequencing data. *Genome Biol*, **11**, R39.

27. Sadeghi, B., Ahmadi, H., Azimzadeh-Jamalkandi, S., Nassiri, M.R. and Masoudi-Nejad, A. (2014) BosFinder: a novell pre-microRNA gene prediction algorithm in Bos taurus. *Animal genetics*.

28. Szcześniak, M.W., Deorowicz, S., Gapski, J., Kaczyński, Ł. and Makałowska, I. (2012) miRNEST database: an integrative approach in microRNA search and annotation. *Nucleic acids research*, **40**, D198-D204.

29. Szcześniak, M.W. and Izabela, M. (2014) miRNEST 2.0: a database of plant and animal microRNAs. *Nucleic acids research*, **42**, D74-D77.

30. Landgraf, P., Rusu, M., Sheridan, R., Sewer, A., Iovino, N., Aravin, A., Pfeffer, S.b., Rice, A., Kamphorst, A.O. and Landthaler, M. (2007) A mammalian microRNA expression atlas based on small RNA library sequencing. *Cell*, **129**, 1401-1414.

31. Huang, J.C., Babak, T., Corson, T.W., Chua, G., Khan, S., Gallie, B.L., Hughes, T.R., Blencowe, B.J., Frey, B.J. and Morris, Q.D. (2007) Using expression profiling data to identify human microRNA targets. *Nature methods*, **4**, 1045-1049.

32. Severin, J., Waterhouse, A.M., Kawaji, H., Lassmann, T., van Nimwegen, E., Balwierz, P.J., de Hoon, M.J., Hume, D.A., Carninci, P. and Hayashizaki, Y. (2009) FANTOM4 EdgeExpressDB: an integrated database of promoters, genes, microRNAs, expression dynamics and regulatory interactions. *Genome Biol*, **10**, R39.

33. Hausser, J., Berninger, P., Rodak, C., Jantscher, Y., Wirth, S. and Zavolan, M. (2009) MirZ: an integrated microRNA expression atlas and target prediction resource. *Nucleic acids research*, **37**, W266-W272.

34. Wang, W.-C., Lin, F.-M., Chang, W.-C., Lin, K.-Y., Huang, H.-D. and Lin, N.-S. (2009) miRExpress: analyzing high-throughput sequencing data for profiling microRNA expression. *BMC bioinformatics*, **10**, 328.

35. Nam, S., Li, M., Choi, K., Balch, C., Kim, S. and Nephew, K.P. (2009) MicroRNA and mRNA integrated analysis (MMIA): a web tool for examining biological functions of microRNA expression. *Nucleic acids research*, **37**, W356-W362.

36. Karali, M., Peluso, I., Gennarino, V.A., Bilio, M., Verde, R., Lago, G., Dollé, P. and Banfi, S. (2010) miRNeye: a microRNA expression atlas of the mouse eye. *BMC genomics*, **11**, 715.

37. Ritchie, W., Flamant, S. and Rasko, J.E.J. (2010) mimiRNA: a microRNA expression profiler and classification resource designed to identify functional correlations between microRNAs and their targets. *Bioinformatics*, **26**, 223-227.

38. Bartonicek, N. and Enright, A.J. (2010) SylArray: a web server for automated detection of miRNA effects from expression data. *Bioinformatics*, **26**, 2900-2901.

39. Sarver, A.L., Phalak, R., Thayanithy, V. and Subramanian, S. (2010) S-MED: sarcoma microRNA expression database. *Laboratory investigation*, **90**, 753-761.

40. Sarver, A.L., French, A.J., Borralho, P.M., Thayanithy, V., Oberg, A.L., Silverstein, K.A.T., Morlan, B.W., Riska, S.M., Boardman, L.A. and Cunningham, J.M. (2009) Human colon cancer profiles show differential microRNA expression depending on mismatch repair status and are characteristic of undifferentiated proliferative states. *BMC cancer*, **9**, 401.

41. Gennarino, V.A., Sardiello, M., Mutarelli, M., Dharmalingam, G., Maselli, V., Lago, G. and Banfi, S. (2011) HOCTAR database: a unique resource for microRNA target prediction. *Gene*, **480**, 51-58.

42. Mestdagh, P., Lefever, S., Pattyn, F., Ridzon, D., Fredlund, E., Fieuw, A., Ongenaert, M., Vermeulen, J., De Paepe, A. and Wong, L. (2011) The microRNA body map: dissecting microRNA function through integrative genomics. *Nucleic acids research*, **39**, e136-e136.

43. Burge, S.W., Daub, J., Eberhardt, R., Tate, J., Barquist, L., Nawrocki, E.P., Eddy, S.R., Gardner, P.P. and Bateman, A. (2013) Rfam 11.0: 10 years of RNA families. *Nucleic acids research*, gks1005.

44. Stocks, M.B., Moxon, S., Mapleson, D., Woolfenden, H.C., Mohorianu, I., Folkes, L., Schwach, F., Dalmay, T. and Moulton, V. (2012) The UEA sRNA workbench: a suite of tools for analysing and visualizing next generation sequencing microRNA and small RNA datasets. *Bioinformatics*, **28**, 2059-2061.

45. Lim, L.P., Glasner, M.E., Yekta, S., Burge, C.B. and Bartel, D.P. (2003) Vertebrate microRNA genes. *Science*, **299**, 1540-1540.

46. Lim, L.P., Lau, N.C., Weinstein, E.G., Abdelhakim, A., Yekta, S., Rhoades, M.W., Burge, C.B. and Bartel, D.P. (2003) The microRNAs of Caenorhabditis elegans. *Genes & development*, **17**, 991-1008.

47. Xue, C., Li, F., He, T., Liu, G.-P., Li, Y. and Zhang, X. (2005) Classification of real and pseudo microRNA precursors using local structure-sequence features and support vector machine. *BMC bioinformatics*, **6**, 310.

48. Megraw, M., Sethupathy, P., Corda, B. and Hatzigeorgiou, A.G. (2007) miRGen: a database for the study of animal microRNA genomic organization and function. *Nucleic acids research*, **35**, D149-D155.

49. Alexiou, P., Vergoulis, T., Gleditzsch, M., Prekas, G., Dalamagas, T., Megraw, M., Grosse, I., Sellis, T. and Hatzigeorgiou, A.G. (2009) miRGen 2.0: a database of microRNA genomic information and regulation. *Nucleic acids research*, gkp888.

50. Antonov, A.V., Dietmann, S., Wong, P., Lutter, D. and Mewes, H.W. (2009) GeneSet2miRNA: finding the signature of cooperative miRNA activities in the gene lists. *Nucleic acids research*, **37**, W323-W328.

51. Jacobsen, A., Krogh, A., Kauppinen, S. and Lindow, M. (2010) miRMaid: a unified programming interface for microRNA data resources. *BMC bioinformatics*, **11**, 29.

52. Lu, M., Shi, B., Wang, J., Cao, Q. and Cui, Q. (2010) TAM: a method for enrichment and depletion analysis of a microRNA category in a list of microRNAs. *BMC bioinformatics*, **11**, 419.

53. Naeem, H., Küffner, R., Csaba, G. and Zimmer, R. (2010) miRSel: automated extraction of associations between microRNAs and genes from the biomedical literature. *BMC bioinformatics*, **11**, 135.

54. Bhartiya, D., Laddha, S.V., Mukhopadhyay, A. and Scaria, V. (2011) miRvar: A comprehensive database for genomic variations in microRNAs. *Human mutation*, **32**, E2226-E2245.

55. Kiezun, A., Artzi, S., Modai, S., Volk, N., Isakov, O. and Shomron, N. (2012) miRviewer: a multispecies microRNA homologous viewer. *BMC research notes*, **5**, 92.

56. Antonov, A.V., Knight, R.A., Melino, G., Barlev, N.A. and Tsvetkov, P.O. (2013) MIRUMIR: an online tool to test microRNAs as biomarkers to predict survival in cancer using multiple clinical data sets. *Cell death and differentiation*, **20**, 367.

57. Dong, L., Luo, M., Wang, F., Zhang, J., Li, T. and Yu, J. (2013) TUMIR: an experimentally supported database of microRNA deregulation in various cancers. *J. Clinical Bioinformatics*, **3**, 7.

58. Ahmed, F., Kaundal, R. and Raghava, G.P.S. (2013) PHDcleav: a SVM based method for predicting human Dicer cleavage sites using sequence and secondary structure of miRNA precursors. *BMC bioinformatics*, **14**, S9.

59. Xie, P., Liu, Y., Li, Y., Zhang, M.Q. and Wang, X. (2014) MIROR: a method for cell-type specific microRNA occupancy rate prediction. *Molecular BioSystems*, **10**, 1377-1384.
