# Supplementary material for: Bioinformatic tools for microRNA dissection
Source: Nucleic Acids Res. 2015 Nov 17;44(1):24–44. doi: 10.1093/nar/gkv1221 (PMC4705652; doi:10.1093/nar/gkv1221)
Supplement: SUPPLEMENTARY DATA [file supp_gkv1221_nar-03711-survey-d-2014-File008.pdf]

Supporting material S4 :

Table shows the year of release of each miRBase version

MirBase release	Year
1.1	January 2003
1.2	April 2003
1.3	May 2003
1.4	July 2003
1.5	July 2003
2.0	July 2003
2.1	September 2003
2.2	November 2003
3.0	January 2004
3.1	April 2004
4.0	January 2004
5.0	September 2004
5.1	December 2004
6.0	April 2005
7.0	June 2005
7.1	October 2005
8.0	February 2006
8.1	May 2006
8.2	July 2006
9.0	October 2006
9.1	February 2007
9.2	May 2007
10.0	August 2007
10.1	December 2007
11.0	April 2008
12.0	September 2008
13.0	March 2009
14	September 2009
15	April 2010
16	August 2010
17	April 2011
18	November 2011
19	August 2012
20	June 2013
21	June 2014

This table has been adopted from Van Peer el al (1)

References

1. Van Peer, G., Lefever, S., Anckaert, J., Beckers, A., Rihani, A., Van Goethem, A., Volders, P.-J., Zeka, F., Ongenaert, M. and Mestdagh, P. (2014) miRBase Tracker: keeping track of microRNA annotation changes. *Database*, **2014**, 1-8.
